# RNA silencing and HIV: A hypothesis for the etiology of the severe combined immunodeficiency induced by the virus

**DOI:** 10.1186/1742-4690-5-79

**Published:** 2008-09-11

**Authors:** Linda B Ludwig

**Affiliations:** 1 861 Main Street, East Aurora, New York, 14052, USA

## Abstract

A novel intrinsic HIV-1 antisense gene was previously described with RNA initiating from the region of an HIV-1 antisense initiator promoter element (HIVaINR). The antisense RNA is exactly complementary to HIV-1 sense RNA and capable of forming ~400 base-pair (bp) duplex RNA in the region of the long terminal repeat (LTR) spanning the beginning portion of TAR in the repeat (R) region and extending through the U3 region. Duplex or double-stranded RNA of several hundred nucleotides in length is a key initiating element of RNA interference (RNAi) in several species. This HIVaINR antisense RNA is also capable of forming multiple stem-loop or hairpin-like secondary structures by M-fold analysis, with at least one that perfectly fits the criteria for a microRNA (miRNA) precursor. MicroRNAs (miRNAs) interact in a sequence-specific manner with target messenger RNAs (mRNAs) to induce either cleavage of the message or impede translation. Human mRNA targets of the predicted HIVaINR antisense RNA (HAA) microRNAs include mRNA for the human interleukin-2 receptor gamma chain (IL-2RG), also called the common gamma (γc) receptor chain, because it is an integral part of 6 receptors mediating interleukin signalling (IL-2R, IL-4R, IL-7R, IL-9R, IL-15R and IL-21R). Other potential human mRNA targets include interleukin-15 (IL-15) mRNA, the fragile × mental retardation protein (FMRP) mRNA, and the IL-1 receptor-associated kinase 1 (IRAK1) mRNA, amongst others. Thus the proposed intrinsic HIVaINR antisense RNA microRNAs (HAAmiRNAs) of the human immunodeficiency virus form complementary targets with mRNAs of a key human gene in adaptive immunity, the IL-2Rγc, in which genetic defects are known to cause an X-linked severe combined immunodeficiency syndrome (X-SCID), as well as mRNAs of genes important in innate immunity. A new model of intrinsic RNA silencing induced by the HIVaINR antisense RNA in the absence of Tat is proposed, with elements suggestive of both small interfering RNA (siRNA) and miRNA.

## Background

In life, timing is everything. Developmental transitions must be exquisitely and appropriately timed, for an animal to develop normally. Genes have to know when to turn on and when to turn off. Proteins need to be translated efficiently when (and where) they will do the most good. Two early examples of a unique form of regulation of gene expression by RNA instead of the more usual protein were mediated by the 22-nucleotide *lin-4 *RNA[1,2] and the 21-nucleotide (nt) *let-7 *RNA [3]. These small RNAs were found to regulate the timing of development in the roundworm, the nematode *Caenorhabditis elegans *[1,4,5]. The *lin-4 *22 nt and 61 nt precursor were noted to have antisense complementarity to several sites in the *lin-14 *gene, in sites already known to be important in mediating repression of *lin-14 *[1,2]. Each final, small RNA is processed from larger RNAs and is very specific in its action because it is complementary to sequences in the 3' untranslated regions (3'UTR) of a specific set of mRNAs of protein-coding target genes [1-3,5,6]. Remarkably, the 21 nt RNA encoded by the *let-7 *gene appears to be conserved across species, from the original roundworms and molluscs to drosophila and to vertebrates, including humans [4]. Recently, these tiny RNAs or microRNAs (miRNAs) have been shown to regulate a wide range of biological processes besides developmental timing, including apoptosis, differentiation, hormone secretion, and even cancer (reviewed in [7-11]. It has also been proposed that small RNAs may play important roles in the host-pathogen interaction: both by mammalian cells to defend against viral infections, and by some viruses, in turn, to escape or adapt to RNA silencing [12].

In the human immunodeficiency virus (HIV-1), the virus has already "borrowed" known transcription factor binding sites and enhancer elements (NFAT, NFkB, Tata box, Sp1 sites) to enable it to effectively utilize the human host cell proteins and RNA polymerase in transcription of its mRNAs and genomic RNA[13]. It is perhaps not surprising that it would also make an antisense RNA to enable a mechanism for fine-tuning the timing of final translational expression of its genes [14]. It would be extremely inefficient to make proteins required for the complete virion if conditions in the cell are suboptimal. In the absence of Tat protein, one of the early regulatory proteins made by the virus, short transcripts of approximately 55–60 nt are predominantly observed [15]. Some early experiments even suggested negative regulatory elements or an inducer of short transcripts to maintain the virus in latency, as when the human host cell was not activated [16]. More recent papers have suggested that the trans-activation-response region (TAR) of HIV-1 mRNA, present in all sense HIV-1 transcripts, functions as a microRNA precursor [17,18].

This paper explores the possibility that HIV-1 might incorporate two mechanisms for RNA silencing that contribute to maintenance of a quiescent state in the host cell, in the absence of Tat protein. It was previously shown that the antisense RNA originating from the region of the HIV antisense initiator (HIVaINR) promoter element is produced simultaneously along with the sense transcripts [14]. This HIVaINR antisense RNA forms an intrinsic bimolecular duplex with U3R sense mRNA (at the 3' end of HIV genomic RNA or mRNA) and suggests the capacity for RNA interference (RNAi). The RNAi pathway begins with long double-stranded RNA, which are naturally generated within the host cell from both HIV-1 sense and antisense transcripts [14]. HIVaINR antisense RNA begins off a site in the R region and extends through the U3 region with perfect complementarity but opposite polarity to its template sense DNA U3R strand. It would therefore have perfect complementarity to any sense HIV mRNA consisting of 3' U3R sequence. It also would have perfect complementarity to the beginning region of all HIV-1 sense mRNAs at the 5'R or TAR region, forming a 25 bp duplex as previously described [14]. In the RNAi pathway, double-stranded RNA is processed by Dicer and then unwound into many ≈22 nt small interfering RNAs (siRNAs), with one strand of the duplex small RNA incorporated into a ribonucleoprotein complex called the RNA-induced silencing complex (RISC) [19-22]. Complementary base-pairing between the siRNA incorporated into the RISC and the mRNA determines the targeted mRNA sites, with cleavage of the mRNA directed between the nucleotides pairing to residues 10 and 11 of the siRNA [23,24]. In HIV-1, the siRNAs would be capable of targeting multiple intrinsic sites on HIV mRNAs because of the extensive perfect complementarity of an intrinsically produced HIVaINR antisense RNA. The converse may also be true, inasmuch as the sense strand of the siRNA duplex could also be targeting the HIVaINR antisense RNA.

However, the HIVaINR antisense RNA itself also has extensive secondary structure and is capable of forming intramolecular duplex structures or extended hairpins (discussed below). Some of these intrinsic HIVaINR antisense RNA hairpins fit criteria for a microRNA precursor. Thus, a second mechanism employed by the virus for gene silencing may involve the microRNA (miRNA) pathway utilizing this HIVaINR antisense RNA, which will be explored below. Because of the human gene mRNAs also potentially targeted, this may represent intrinsic mechanisms for (self) viral and human host gene regulation by the HIV-1 virus. In the process, the HIV-1 targeting of specific human genes may have profound effects on the human host adaptive and innate immunity.

## Results and Discussion

### Could the HIVaINR antisense gene encode its own microRNA subspecies?

The capacity for an intrinsic RNA regulatory mechanism for control of HIV-1 gene expression by means of an antisense RNA initiated from the HIVaINR in TAR (LTR) DNA has been suggested previously [14]. This antisense RNA most notably has the capacity to form a duplex of 25 bp with the 5' end of all sense HIV mRNA and genomic HIV-1 RNA (see additional file 3 (figure 3S) in [14]). At the time this was initially proposed in 1996, the known models for duplex RNAs regulating genes were in prokaryotes [25,26]; the term "microRNA" would not be coined until 2001 [6,27,28]. However, this same HIVaINR antisense RNA which encodes antisense proteins (HAPs), also has the capacity to form hairpin structures that could be precursors to the formation of intrinsic viral microRNAs (vmiRNAs) named the HIVaINR antisense RNA miRNAs or HAAmiRNAs [14]. Others have suggested the possibility for HIV microRNAs encoded by the sense strand of HIV mRNAs with the potential for an entirely different set of human cellular target mRNAs[17,18,29,30].

**Figure 1:**
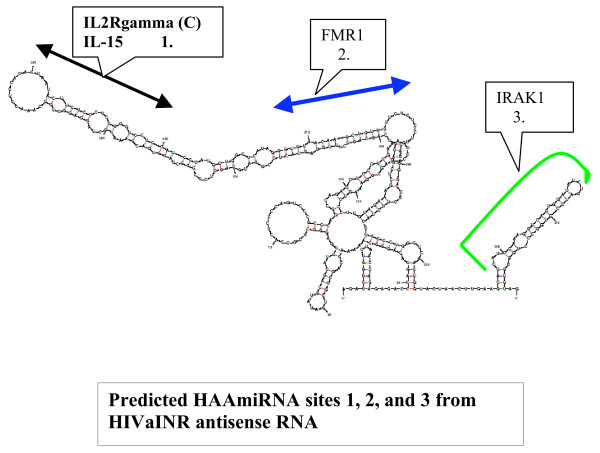
**Secondary structure of HIVaINR antisense RNA**[14]** predicted by enhanced Mfold **[31-33]. This is one of 19 structures predicted, but was chosen to illustrate the extensive duplex structure of the HIVaINR antisense RNA[14], with the predicted microRNA sites 1, 2, and 3 indicated. HAAmiRNA site 1 has complementary sequence to multiple sites in mRNA of the interleukin-2 receptor (IL-2R) gamma chain, also called the common γ chain, and to sites in the mRNA of interleukin-15 (IL-15). HAAmiRNA site 2 has complementary sequence to fragile-X mental retardation protein (FMR1) mRNA. HAAmiRNA site 3 has complementary sequence to sites in the interleukin-1 (IL-1) receptor-associated kinase 1 (IRAK1) mRNA. Discussed in text.

HIVaINR antisense RNA forms extensive intrinsic duplex structure by M-fold analysis and DINAMelt server (see Figure 1 and additional file 1). Nineteen separate HIVaINR antisense RNA duplex structures with dG of -99.2 to -94.9 could form by the enhanced Mfold program (additional file 1) [31-33]. The plasticity of structure demonstrated is remarkable, but still does not represent all the potential influences on 3-dimensional RNA structure; the effect of protein binding or pseudoknot formation is not considered. miRNAs are generated from long primary transcripts containing hairpin or stem-loop structures (pri-miRNAs) that are first processed in the nucleus by the RNase III enzyme Drosha in partnership with the dsRNA binding protein, DGCR8 or DiGeorge syndrome critical region gene 8 [34-36]. The prototypic metazoan pri-miRNA consists of a stem of ~32–33 base-pairs (bp) with a terminal loop and flanking single-stranded RNA at the base of the stem-loop, although in plants, the stem-loop might be much longer [7,37]. Cleavage by the Drosha-DGCR8 complex converts the pri-miRNA into small stem-loop structures called precursor miRNAs (pre-miRNAs). This is then further processed by another RNase III enzyme (Dicer)/dsRNA binding protein duo into mature miRNAs. In an elegant paper by Ritchie, et al., they addressed what parameters might distinguish precursor miRNAs (pre-miRNAs) from other duplex structures of similar size and free energy [38]. In a cellular world in which long RNA duplexes are frequent, the RNAse III enzymes of the microRNA pathways, Drosha and Dicer, must be able to distinguish the appropriate RNA stem-loops that signal a primary or precursor miRNA for cleaving into the mature 21- to 25- nucleotide (nt) long, single-stranded miRNA [38,39]. Some reports suggest that a larger apical loop size, as well as flanking single-stranded RNA extensions at the base of the primary miRNA hairpin is important for Drosha function[40,41]. A recent study found the terminal loop was not essential, but the cleavage site for Drosha was determined by the distance (~11 bp) from the base of the hairpin stem and single-stranded RNA junction [37].

**Figure 2:**
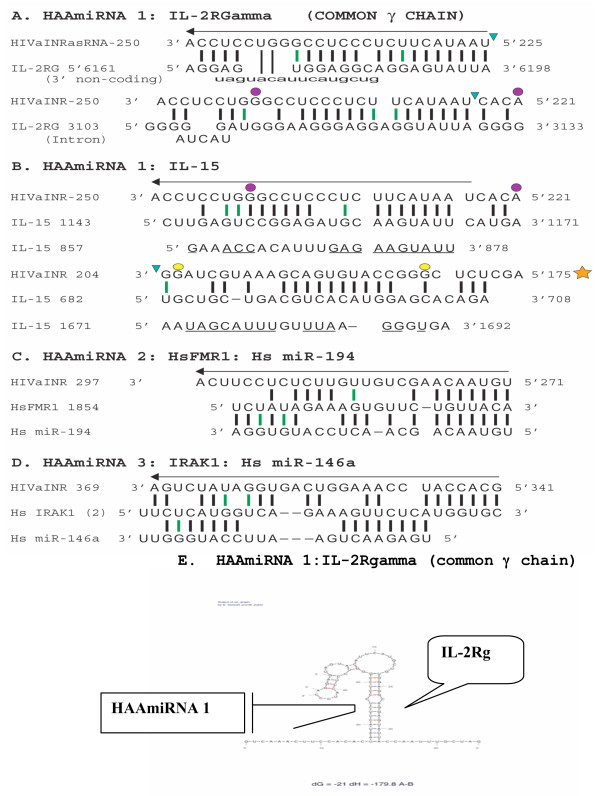
**Proposed HAAmiRNA human target genes**. (A) Complementary base-pairing between the HIVaINR antisense RNA site 1 (HAAmiRNA1) from nucleotides (nt) ~225–250 [14] and mRNA sequence encoding the interleukin-2 receptor gamma chain (IL-2RG or γC) from nt 6161–6198 in the 3' UTR (upper) and from nt 3103–3133 in an intronic region (lower). The human IL-2RG sequence was obtained from the NCBI GenBank AY692262. (B) Both strands of HAAmiRNA 1,1* target complementary sites in human interleukin-15 (IL-15) mRNA. HAAmiRNA 1 nt 225–250 [14] target IL-15 nt 1146–1166 and IL-15 nt 857–878, underlined (upper). The opposite strand HAAmiRNA 1* (yellow star) also targets sites in IL-15 mRNA, as indicated. IL-15 sequence is GenBank NM172174 transcript variant 1. Purple dots indicate proposed siRNA sequence. (C) HAAmiRNA site 2 from nt 271–297 [14] complementary base-pairing to human fragile × mental retardation protein mRNA (HsFMR1) is compared with the interaction between HsFMR1 and human miRNA-194 [48]. (D) HAAmiRNA site 3 from nt 341–369 [14] complementary base-pairing to human interleukin-1 (IL-1) receptor-associated kinase 1 (IRAK1) mRNA at site 2 is compared to human miRNA-146a [77]. (E) The Mfold structure formed between HAAmiRNA1 and IL-2RG mRNA [31-33].

While folding free energy and stem length were not sufficient to discrimate miRNA precursors from other long RNA duplexes, it was determined by computational analysis that nonprecursor duplexes differed from real miRNA precursors in having increased lengths and numbers of bulges and internal loops and larger apical loop size [38]. These secondary structure characteristics were utilized in developing a miRNA prediction algorithm, with comparisons done using the RNAforester tool [42,43]. When the HIVaINR antisense RNA sequence from nt 168–253 [14] was submitted to this structure-based miRNA analysis tool for analysis, it received a perfect score (100) consistent with this sequence being a microRNA precursor (Ritchie et al, ) [38]. Further comparison with the M-fold duplexes demonstrated that even with the 390 nucleotide HIVaINR antisense RNA [14] subjected to enhanced M-fold, some of the structures could potentially be processed (first by Drosha, then Dicer) into this final pre-miRNA (see additional file 1, structure with folding energy dG = -96.7). This was important, inasmuch as the HIVaINR antisense RNA stem-loop also contained 25 bases that could in turn form yet another duplex or target with several human mRNAs. Two of the many mRNAs targeted included mRNA of the human gene, interleukin-2 receptor gamma chain (IL-2Rγc), a gene in which defects are responsible for X-linked severe combined immuno-deficiency (X-SCID), as well as the human interleukin-15 mRNA, discussed below (diagrammed in Figure 2A, B, E).

### Human interleukin-15 mRNA: a proposed target of the HIVaINR antisense RNA site 1 (HAAmiRNA 1, *1)

HIVaINR antisense RNA sequence from nt 168–253 [14] is capable of forming a stem-loop or hairpin structure consistent with a precursor miRNA (Figure 1 and additional file 1) [31,33,38]. The hairpin structure or pre-miRNA thus could be processed by Dicer to yield two strands of short RNA. Each strand appears capable of interacting with a number of human target mRNAs using BLASTN of the NCBI (Figure 1, Figure 2, and data not shown). In microRNAs, a core element or "seed" region of ~7 or 8 nucleotides (nt) at the 5' region of the microRNA is particularly required for microRNA complementary base-pairing to the messenger RNA (mRNA) target sequences[44]. Residues 2–8 of the microRNA have been proposed to represent the core region initially presented by the RNA-induced silencing complex or RISC for nucleate pairing to the mRNAs (reviewed in [7,39]). If sufficient additional base-pairing between the microRNA and mRNA occurs, cleavage of the message (mRNA) can occur [7]. However, the core "seed" pairing, supplemented by just a few flanking base-pairing residues appears sufficient to mediate translational repression[7,45].

Figure 2B illustrates some of the interactions possible between the HAAmiRNA site 1 strands and human mRNA for interleukin-15 (IL-15). HAAmiRNA site 1 from nucleotides 225–246[14] can form a complementary base-paired structure with human IL-15 mRNA at multiple sites. Interleukin-15 mRNA nucleotides 1143–1171 and HAAmiRNA 1 form a duplex with 19 base-paired elements, including a 7 base-pair "seed" (Figure 2B). IL-15 mRNA from nucleotides 857–878 and HAAmiRNA 1 form a duplex with 14 base-pairs, including a 10 base-pair "seed" (Figure 2B, underlined). The opposing strand of the precursor miRNA (HAAmiRNA 1*) might also target human IL-15 mRNA (Figure 2B, yellow star). HAAmiRNA 1* from nucleotides 175–204 [14] forms a 18 base-pair duplex with human IL-15 mRNA nucleotides 682–708, including a 10 base-pair "seed" (Figure 2B, yellow star). It is not unusual that a functional microRNA will target multiple sites in a mRNA [44,46,47]. It is interesting that several of these target sites are in the IL-15 mRNA coding region, which is expected in plants, but has typically not been looked for in mammals, where the focus has been on detecting target sites in the 3'UTR [48]. However, it has been reported that short RNAs partially complementary to a single site in the coding sequence of mRNA targets of endogenous human genes can mediate translational repression [49]. Given viral versatility and adaptability, it would be premature to assume that only the 3'UTR of mRNAs could be the target for vmiRNAs.

Interleukin-15 is a cytokine that is important in regulation of T-cell maturation and natural killer (NK) cell development and that is secreted by human macrophages and other cells [50-54]. Interleukin-15 and interleukin-7 are required for survival of long-lived memory T cells [50,55]. Studies in mice and humans suggest that a functional IL-15/IL-15 receptor signalling pathway is required for development and survival of NK cells [50-52,54]. NK cells are a class of lymphoid cells that contribute to innate host defense against intracellular pathogens and viruses, as well as tumor cells[54]. The IL-15 receptor consists of a unique IL-15Rα chain that combines with two other receptor chains that are also shared with the IL-2 receptor, the β and γc subunits. HAAmiRNA 1 also potentially targets the γc subunit or IL-2Rgamma chain mRNA (discussed below). The combined effects of HIV-1 microRNA action to inhibit protein production from these mRNAs would be predicted to impact on natural killer cell function. Because NK cell activity represents one of the early host innate immune responses against virally infected cells, HIV-1 could thereby strike an early and crippling blow against the human immune response.

### Interleukin-2 receptor gamma chain (common gamma chain) (γC)- a proposed human mRNA target of the HIVaINR antisense RNA site 1 (HAAmiRNA 1, 1*)

The HIVaINR antisense RNA stem-loop precursor (HAAmiRNA 1,1*) also contains sequence that can form duplex structures with several sites on mRNA encoding the interleukin-2 receptor gamma chain (IL-2RG). IL-2RG is now known as the common gamma (γc) cytokine receptor chain because it is a component of the interleukin receptors IL-2R, IL- 4R, IL-7R, IL-9R, IL- 15R, and IL-21R [56-59]. Genetic defects or mutations in IL-2RG (γc) gene can cause X-linked severe combined immunodeficiency (X-SCID) secondary to the profound T cell and NK cell deficiency induced by lack of a functional γc gene [60-62] X-linked SCID is so severe that some children who inherit it can only survive following bone marrow transplantation or in a pathogen-free environment, as demonstrated by the Houston child, the "boy in the bubble".

HAAmiRNA 1 from nt 225–250[14] is involved in extensive complementary base-pairing to several sites in the 3' UTR of IL-2RG mRNA as well as to sites in intronic and 5' regions of the IL-2RG mRNA (Figure 2A, and data not shown). HAAmiRNA 1 interaction with the 3'UTR of IL-2RG mRNA is illustrated in Figure 2A and Figure 2E. A critical 5' seed of 10 nt are base-paired, followed by a bulge at nt 11, followed by 11 more interrupted sites of base-pairing such that 22/25 nt of the HAAmiRNA 1 is base-paired to the target site (Figure 2A, 2E). HAAmiRNA 1 targets a complementary site in an intron of IL-2RG, with 21 out of 26 nt potentially base-paired with the intronic site (Figure 2A, lower). Interestingly, if Dicer cuts the intermolecular duplex formed by both HIV-1 sense RNA and HIVaINR antisense RNA, one of the predicted ~22 nt cleaved fragments (siRNAs) would contain the overlapping sequence from nt 221–242 [14] (indicated by purple dots in Figure 2A, 2B).

### If the human immunodeficiency virus wanted to turn off T-cell proliferation to enable it to subvert the cell's machinery for other purposes, IL-2RG chain (γc) would be the perfect switch

The adaptive immune response requires appropriate co-signals and cytokine stimulation for the T cell to proliferate in response to recognition of a specific antigen. This is one of the defining aspects of adaptive immunity: the capacity to greatly expand the population of T-cells (or B cells) that specifically recognize a foreign antigen and thereby bring an infection under control. Central to this pathway activating lymphocyte proliferation and, paradoxically, lymphocyte death is an autocrine (and paracrine) loop involving interleukin-2 and the tripartite interleukin-2 receptor complex, IL2-R[63]. The interleukin-2 receptor (IL2-R) and IL-15 R are heterotrimers that consist of a unique α-chain but share the IL-2R gamma (γ common or γc) chain and IL-2Rβ chain [59,63,64]. The receptors for the interleukins IL-4, IL-7, IL-9, and IL-21 are heterodimers with unique α-subunits and the shared subunit, IL-2RG or γc chain [56-58,64]. For all of these cytokine receptors, the γc chain contributes to ligand binding as well as signal transduction within the cell [54,64-66].

Targeting the human lymphoid cell IL-2Rgamma chain or γc mRNA by HAAmiRNA 1 could lead to multiple changes within the cell: impaired production of this receptor chain protein could alter or eliminate the human CD4+ T cells ability to proliferate and mount an effective adaptive immune response and also impair NK cell functioning via the IL-15R and IL-21R with an impact on innate immunity[56]. NK function also could be impacted by the absence of a critical cytokine, IL-15, discussed above. Mutation or gene deletion of the IL-2Rgamma chain (γc) in humans causes extremely low numbers of T cells, poor or absent T cell mitogen responses, severely depressed NK cell function, and an elevated or normal proportion of B cells that fail to produce specific antibodies[62]. Each of these defects are observable in the immune system of HIV-infected individuals, even before significant depletion of CD4+ T cells[67]. Even a single missense mutation in the γc chain can lead to a progressive T cell deficiency [68]. By analogy, one might expect the gradual accrual over time of a similar phenotype, as more human cells are infected by the virus and then incapacitated by viral microRNA translational inhibition (or cleavage) of the γc mRNA.

### Other HIVaINR antisense RNA predicted miRNAs?

A variety of strategies were used to identify potential miRNA sites within the HIVaINR antisense RNA sequence [14]. First, the identification of potential microRNA precursor sites on the HIV-1 sense RNA strand immediately suggested similar structures were possible on the corresponding complementary antisense RNA that overlapped these regions [29,30] (diagrammed in additional file 2). This provided the impetus to look at sites encompassing HAAmiRNAs 1 and 3 (Figure 2A, 2B, and 2D). Second, the miRNA prediction algorithm, described by Ritchie et al ) [38] was also utililized to analyze overlapping sets of the HIVaINR antisense RNA sequence. Third, because the entire 390 nt sequence [14] was also analyzed by enhanced M-fold [31-33], visual inspection of the RNA duplexes formed was possible, with extrapolation of potential cleavage sites by Drosha and Dicer. For instance, if Drosha-DGCR8 complex requires a minimum of 33 bp stem structure in conjunction with unpaired or single-stranded RNA at the base of the stem [37], then a single very extensive hairpin in the structure labeled dG = -96.7 HAAmiRNA, additional file 1, provides a substrate that could be cleaved to release potentially both HAAmiRNAs 1 and 2. The M-fold analysis of the much longer sequence also provided insight into potential cleavage sites that would not be detected using analysis simply of contiguous 80–100 nucleotide sequences. Fourth, a vmiRNA of interest might have complementary human mRNA targets (as illustrated with HAAmiRNA 1, 1*).

HIVaINR antisense RNA at site 2 between nucleotides 271–297 could potentially target mRNA sequence for human fragile × mental retardation protein (FMRP) (Figure 1, Figure 2C). Key aspects of miRNA and target mRNA matches are: 1) the 5'end of the miRNA tends to have more bases complementary than the 3' end (with a seed 7 nt base paired in many cases); 2) loopouts in either mRNA or miRNA between miRNA nt 9 and 14 are often observed; 3) G:U wobble base-pairs are less common in the 5' end of the miRNA:mRNA duplex (reviewed in[44,48]). HAAmiRNA 2 interaction with the human mRNA for FMRP meets these criteria (Figure 2C, compare complementary base-pairing between HIVaINR antisense RNA nt 271–297 and HsFMR1)). It is interesting that human microRNA 194 (Hs miR-194) also targets this site in human FMRP mRNA (Figure 2C).

This could be of major impact, if verified experimentally, because not only does the FMRP play a role in protein synthesis and bind large numbers of cellular mRNAs through G-quartet and U-rich motifs [69-72], but experimental evidence links FMRP with RISC components and miRNAs [73-75]. Mammalian FMRP interacts with miRNAs and Dicer and the mammalian orthologues of Argonaute (AGO) 1 [73,75]. Whether HAAmiRNA 2 targeting human mRNA for FMRP results in translational repression or cleavage of the human mRNA for FMRP, the effects will be amplified because of the large number of human mRNAs targeted by the FMRP protein itself. This would enable the virus to immediately impact on many hundreds of cellular messages. In addition, the primary RNAs for human microRNAs may be impacted, as has already been suggested for HIV-1-transfected human cells [30]. HIV potentially could thus use HAAmiRNA 2 to regulate the host effort to demolish the virus through host miRNA/siRNA silencing pathways. If HAAmiRNA 2 impedes efficient translation of FMRP, it also will affect FMRP interaction with proteins of the RNA-induced silencing complex. Others have already shown the importance of the two RNAase III enzymes fundamental to RNA silencing, Drosha and Dicer, in inhibiting HIV replication[76].

HIVaINR antisense RNA site 3 from nt 341–369 [14] (herein referred to as HAAmiRNA 3) could potentially target human mRNA for the interleukin-1 receptor-associated kinase 1 (IRAK1) (Figure 1 and 2D[77]. It targets IRAK1 mRNA via an overlapping site when compared to IRAK1 interaction with the human microRNA, miR-146a (Figure 2D) [77]. Many miRNAs are postulated to act cooperatively for translational repression, requiring two or more target sites per message [48,78]. However, multiple, diverse miRNAs may impinge on multiple target sites within a mRNA, leading to effects of multiplicity or cooperativity that fine-tune translational repression [78]. HAAmiRNA 3 forms a reasonable "seed" structure of 7 complementary base-pairing nucleotides at the 5'end, followed by 12 more complementary base-pairing nucleotides that can encompass the human mRNA IRAK1 site 2 (Figure 2D). This can be compared to human miR-146a, which forms a complementary base-pairing "seed" site utilizing 8 nucleotides at the 5' end of the miRNA, followed by a gap of 5 nucleotides, then 7 nucleotides that base-pair to the human IRAK1 mRNA site 2 [77] (Figure 2D).

It is of particular interest that human miR-146 has been shown to functionally interact with human mRNA 3'UTR sites for IRAK1 and thereby downregulate protein expression[77]. Expression of primary miR-146 transcripts is regulated by NF-kB sites, sites that are also important enhancer elements for expression of HIV-1 RNA transcripts [14,77,79]. IRAK1 is involved in the signalling cascade induced by activation of Toll-like receptors (TLRs) that are important in innate immunity. Experimental evidence that miR-146a/b may function as a novel negative regulator has been recently shown [77]. If HIV uses a microRNA mechanism like miR-146a to interact with IRAK1 mRNAs, which are expressed in macrophages and dendritic cells, it may provide yet another means for early viral impact on the host innate immunity pathways.

### RNA silencing by HIVaINR antisense RNA-a proposed model (Figure 3)

**Figure 3:**
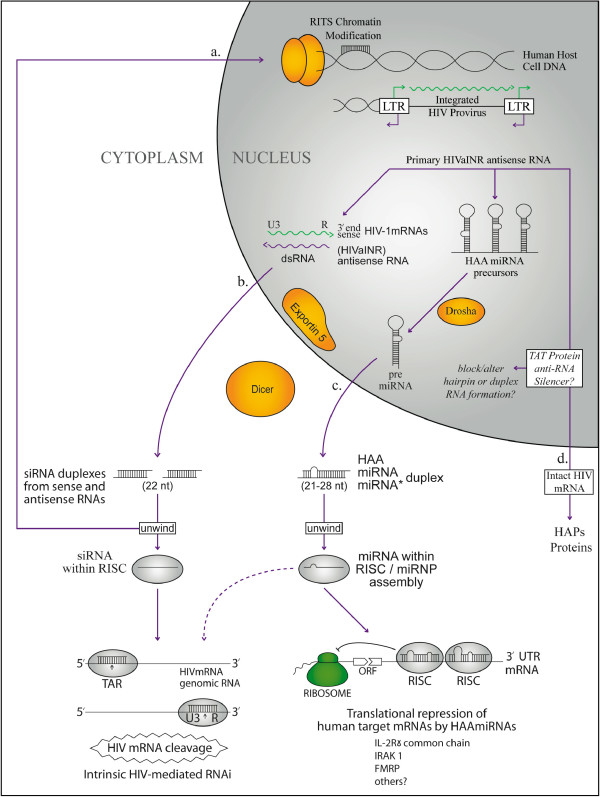
**RNA silencing by HIVaINR antisense RNA**. MicroRNAs (miRNAs) and small interfering RNAs (siRNAs) could be processed from the HIVaINR antisense RNA[14] and duplex RNAs using the host cell protein components of the RNA interference (RNAi) and miRNA pathways. These small RNAs (siRNAs/miRNAs) are proposed to control gene expression in the human host cell in a sequence-specific manner by: (a) chromatin modification and silencing; (b) HIV-mediated RNAi leading to complementary target messenger RNA (mRNA) degradation; (c) miRNA targeted translational repression, and also cleavage if sufficient complementary sequence. (d) Tat protein could function to eliminate or suppress RNA silencing and thereby allow intact mRNA for protein production. Discussed in text.

While RNA silencing triggered by double-stranded RNA [dsRNA] precursors occurs in a wide variety of eukaryotic organisms as a mechanism to regulate gene expression[22], early experiments in plants also suggested RNA silencing is employed as an antiviral mechanism to protect from RNA viruses [80-83]. To survive, viruses have had to evolve mechanisms to suppress or avoid the host RNA silencing response [83,84].

In this model, I propose that HIV-1 employs limited genetic space to best effect by producing a primary HIVaINR antisense RNA with multiple functions (Figure 3a–d)[14]. The HIVaINR antisense RNA encodes a set of proteins called HIV antisense proteins (HAPs) [14]. The same HIVaINR antisense RNA enables intrinsic viral RNA silencing employing short interfering RNAs (siRNAs) and microRNAs (miRNAs) (Figure 3b and 3c). HIV-1 demonstrates versatility because the endogenous HIVaINR antisense RNA transcript originating from the HIV antisense initiator site (HIVaINR) in the long terminal repeat (LTR) of the provirus has the intrinsic capability of being employed in either silencing pathway (Figure 3b and 3c). Because this HIVaINR antisense transcript is produced off of template U3R sequences of the HIV DNA (sense) strand, it is exactly complementary in sequence to the sense HIV mRNA (or HIV genomic RNA) in the U3 (untranslated 3') R (repeat) regions. Thus, hybridization of overlapping transcripts from sense HIV mRNAs (at the U3R 3' end) and the HIVaINR antisense RNA produced from either LTR can form a perfect duplex or double-stranded RNA of 400–450 bp (Figure 3b). This can function as an initiating substrate for the RNA interference (RNAi) pathway and the production of multiple siRNA duplexes by Dicer (Figure 3b). Once each siRNA duplex is unwound and a single 21–22 nucleotide (nt) strand is incorporated into the RNA-induced silencing complex (RISC), it can potentially guide mRNA degradation (Figure 3b) or chromatin modification (Figure 3a). The siRNA interacts in a sequence-specific manner with the corresponding complementary sequences in (sense) HIV mRNA found at the beginning TAR region (5') as well as in multiple sites at the end of all sense mRNA transcripts containing U3R (3') (diagrammed in Figure 3b), as previously described [14,85]. By cleavage of the corresponding sense mRNAs at the many sites available in the exactly complementary regions spanning the U3R-3', and the beginning portion of the TAR mRNA at the 5'end of HIV sense messages, these HIVaINR antisense RNA-generated siRNAs could profoundly impact HIV gene expression.

The endogenous HIVaINR antisense RNA further has the intrinsic capacity for forming multiple dsRNA hairpin structures with complementary or near-complementary base-pairing (Figure 1 and additional file 1). Primary HIVaINR antisense RNA has the potential to form precursor-like microRNAs and HAAmiRNAs, as discussed above (Figure 3c). In mammals, maturation of miRNAs is initiated by nuclear cleavage of longer primary miRNA transcripts by the Drosha RNAse III endonuclease to liberate stem-loop precursors referred to as precursor miRNAs (pre-miRNAs) (Figure 3c)[34,86]. Drosha exists in a complex with a dsRNA-binding protein called DGCR8 [35-37]. This initial cut by Drosha yields a stem-loop with a 5'phosphate and 2 nt 3' overhang at the base [34,87]. Pre-miRNA is then transported out of the nucleus by Ran-GTP and Exportin-5 [88-90], where it would be cleaved by the RNase III enzyme Dicer to form the HAAmiRNA/miRNA* duplex (Figure 3c) [34,91]. Dicer is believed to use a similar mechanism to that proposed for bacterial RNase III to generate ≈22 miRNA duplexes [92]. Dicer functions in both the miRNA maturation pathway and the siRNA generation pathway (Figure 3b and 3c), reviewed in [7,93]. Recently Dicer was shown to operate along with the TRBP or transactivating response RNA binding protein [94-96]. Like the siRNA duplex, the miRNA:miRNA* duplex is unwound, and one miRNA strand is preferentially associated with a ribonucleoprotein complex (miRNP) containing the proteins eIF2C2, and helicases Gemin3 and Gemin4 [85,97,98]. The miRNA within the ribonucleoprotein complex serves to guide the protein machinery to complementary sites in the human cell messenger RNAs (mRNAs), where either translational repression or message cleavage occurs[7,22,78]. It is also possible that the intrinsic HAAmiRNAs might target corresponding targets in the viral mRNA (Figure 3c, dotted arrow). The human Argonaute homolog eIF2C2 is a component of the human siRNA-RNA-induced silencing complex (RISC) [85]. Therefore, the RISC/miRNP components may be similar, if not indistinguishable (Figure 3b, 3c). The "minimal" active RISC may contain only Argonaute (Ago) proteins associated with siRNAs, indicating that the Ago component catalyzes mRNA cleavage[85,99]. In mammals, only Ago2 is able to support mRNA cleavage upon incorporation in the RISC [99-101]. Mutagenesis of recombinant human Ago2 showed that a DDH rather than a DDE triad of amino acids played a critical role in catalysis [101]. In addition to the guide siRNA/miRNA and Ago, the core catalytic component of the RISC [75], additional proteins that have been (variably) associated with the RISC include the Vasa intronic gene protein (VIG), Fragile X-related protein (drosophila) or the human fragile × mental retardation protein (FMRP), and Tudor-SN [22,73-75,102].

Why would a virus utilize host cell enzymes Drosha and Dicer to dice up its own messages? Here, we must return to the initial concept of this paper, where timing of gene expression is so important to viral survival. The early skirmish in the battle between the virus and the host cell might require some sacrifice of intact viral messages for a time. By generating HAA miRNAs that incorporate into the host cell RISC, the virus can impede mRNAs from the host genes critical in enabling host innate as well as adaptive immune responses. The virus thereby employs the host's own anti-viral RNA silencing defence against the host cell. It can't be accidental that HAAmiRNA 1 has sequence that could target multiple complementary sites in human mRNA for the human IL-2R γ or common gamma (γc) chain, a requisite component in the receptors for all known T-cell growth factors (interleukins (IL)-2, IL-4, IL-7, IL-9, IL-15, and IL-21). The same HAAmiRNA 1 sequence can also target multiple sites in the interleukin-15 mRNA, a key cytokine involved in NK cell functioning.

In addition, until conditions are appropriate in the host cell for intact HIV RNA and protein production, dicing up the early transcripts (Figure 3b) or using miRNA/RISC for translational repression (Figure 3c) would prevent host innate and adaptive immune responses from obtaining any sort of head start for recognition of viral proteins. The presence of Tat protein, once the cell is activated, might provide the signal that allows a preponderance of intact viral mRNA to be made for more protein production and production of virions (Figure 3d). It has been proposed that the HIV-1 Tat protein also functions as a suppressor of RNA silencing, by subverting the ability of Dicer to process dsRNAs into siRNAs[84]. In plants, viruses have evolved a variety of mechanisms to suppress RNA silencing [83,103,104]. However, Tat protein also binds directly to the HIVaINR antisense RNA, and alters RNA stability (LBL, unpublished observations). The mechanism for this is unknown. A simple hypothesis would be that Tat protein, through its interaction with the HIVaINR antisense RNA might alter the secondary/tertiary structure of the HIVaINR antisense RNA, such that formation of the required microRNA hairpin precursor(s) is altered and functional microRNA does not result (diagrammed in Figure 3d). Experiments have shown that alteration of RNA secondary structure by mutation can allow HIV-1 to escape RNAi because of occlusion of an siRNA-target sequence [105]. Alternatively, Tat could act at the level of Dicer, as previously suggested[84], or through another mechanism. Regardless, in order to produce the HIV antisense proteins called HAPs, it was necessary to use a Tat-producing cell line (Figure 3d)[14].

### Implications for HIV-1 vaccine development

The particular HIV-1 genetic regions encompassing HAAmiRNA sites 1–3 are well conserved in the B clade, and even more remarkably, particularly conserve miRNA sequence required for mRNA target recognition in most of the clades of group M (A-D, F-H, J and K), with the exception of the O group strains (underlined, additional file 2)[106,107]. There is even conservation of a 7 nucleotide "seed" of the HAAmiRNA1 in some of the chimpanzee virus variants, CPZ.CAM 3 and 5 (additional file 2). The HAAmiRNA site 1 and site 1* region overlaps and is complementary to the predicted #4 microRNA precursor previously described by Bennasser, et al[30], and is bordered by one of the most variable regions in the HIV-1 LTR called the most frequent naturally-occurring length polymorphism (MFNLP) [106]. Conservation of a precursor microRNA would be expected to be in balance with the viral need to escape host RNA silencing mechanisms. The endogenous siRNA produced by the virus, however, mutates along with the viral template. This suggests that selection pressure has maintained the microRNA sites as essential for the virus. Even more interesting is that the precursor microRNA 1 hairpin is entirely deleted/mutated in a group of long-term survivors, who continued with T cell function for longer than expected[108]. If, as suggested in this paper, this particular site targets the human IL-2RG (common gamma chain) mRNA and IL-15 mRNA, and can be shown to impact on T-cell and NK-cell function, this must be taken into consideration when designing vectors for gene therapy. Care must be taken to remove these HAAmiRNA sites or alter their function, if introducing the HIV-1 LTR into susceptible cells. Alternatively, specifically targeting these sites with siRNAs that eliminate their function without perpetuating any genetic damage to the host might be considered.

## Conclusion

RNA silencing for regulation of gene expression is now recognized as an important tool for many species, including humans, to control how and when proteins are made. Viruses have undoubtedly already developed mechanisms that allow them to survive in their host mammalian cell, including subversion of the host cell machinery for RNA silencing. HIV encodes, within its long terminal repeat, an antisense gene responsible for RNA and protein products[14]. The antisense RNA transcribed from this gene can generate an intrinsic, perfectly complementary RNA that base-pairs to the beginning and end portion of the genomic HIV RNA and mRNAs for the viral proteins. Double-stranded RNA initiates RNAi and could allow intrinsic HIV control of when viral RNAs are made. In addition, the antisense RNA forms an intrinsic, intramolecular duplex RNA consistent with microRNA precursor stem-loops. Precursor microRNA stem-loops have already been proposed for the sense HIV-1 RNA[17,18,29,30]. The individual human cellular mRNAs potentially targeted by a single-stranded short RNA (miRNA) derived from this HIV precursor RNA structure turn out to be mRNAs very important in the human adaptive (and innate) immune response. One of the (many) targets of the HIVaINR antisense RNA miRNAs (HAAmiRNAs) is the human interleukin-2 receptor gamma chain, also known as the gamma common chain because it is a component of 6 separate cytokine receptors important in immune cell signalling and interactions. By this mechanism, I propose the human immunodeficiency virus has found a way to cripple effective host cell immune responses. In designing HIV vaccines, this must be taken into account, because incorporation of this HIV antisense gene segment could functionally impair, rather than build an effective immune response. Alternatively, designing gene therapy that targets this HAAmiRNA encoding gene segment, before it gets to the CD4+ T cell, might change the balance of power between virus and human host.

## Competing interests

The author declares that they have no competing interests.

## Authors' contributions

LBL accepts full responsibility for the observations/opinions stated herein.

## Supplementary Material

Additional file 1 HIVaINR antisense RNA [14] analyzed by Mfold [31-33]. Click here for file

Additional file 2 HIVaINR antisense RNA [14] and predicted HAAmiRNAs at sites 1, 2 and 3 (backward yellow arrows) are shown above the corresponding complementary sequence in the HIV-1 sequence alignments for group M (strains A, B, C, D, F1, G, H, J and K), group N, and group O, as well as chimpanzee (CPZ) strains in the long terminal repeat (LTR), as obtained from the HIV Sequence Compendium 2000[107]. HAAmiRNA 3 overlaps and is partially complementary to the nef microRNA:miR-N367 described by Omoto, S., et al., [29] and HAAmiRNA 1 and precursor (pre-HAAmiRNA 1) overlaps and is complementary sequence to the predicted #4 microRNA precursor described by Bennasser, Y. et al [30]. Predicted seed regions for the microRNAs are shaded, and sequence targeting human mRNA is underlined. The B clade HXB2 is the reference sequence, with identical sequences in the strains below, unless otherwise indicated [107]. Click here for file
