# Supplementary material for: RNA silencing and HIV: A hypothesis for the etiology of the severe combined immunodeficiency induced by the virus
Source: Retrovirology. 2008 Sep 11;5:79. doi: 10.1186/1742-4690-5-79 (PMC2553099; doi:10.1186/1742-4690-5-79)
Supplement: Additional file 1 — HIVaINR antisense RNA [14] analyzed by Mfold [31-33]. [file 1742-4690-5-79-S1.pdf]

Output of sir_graph
by D. Stewart and M. Zuker

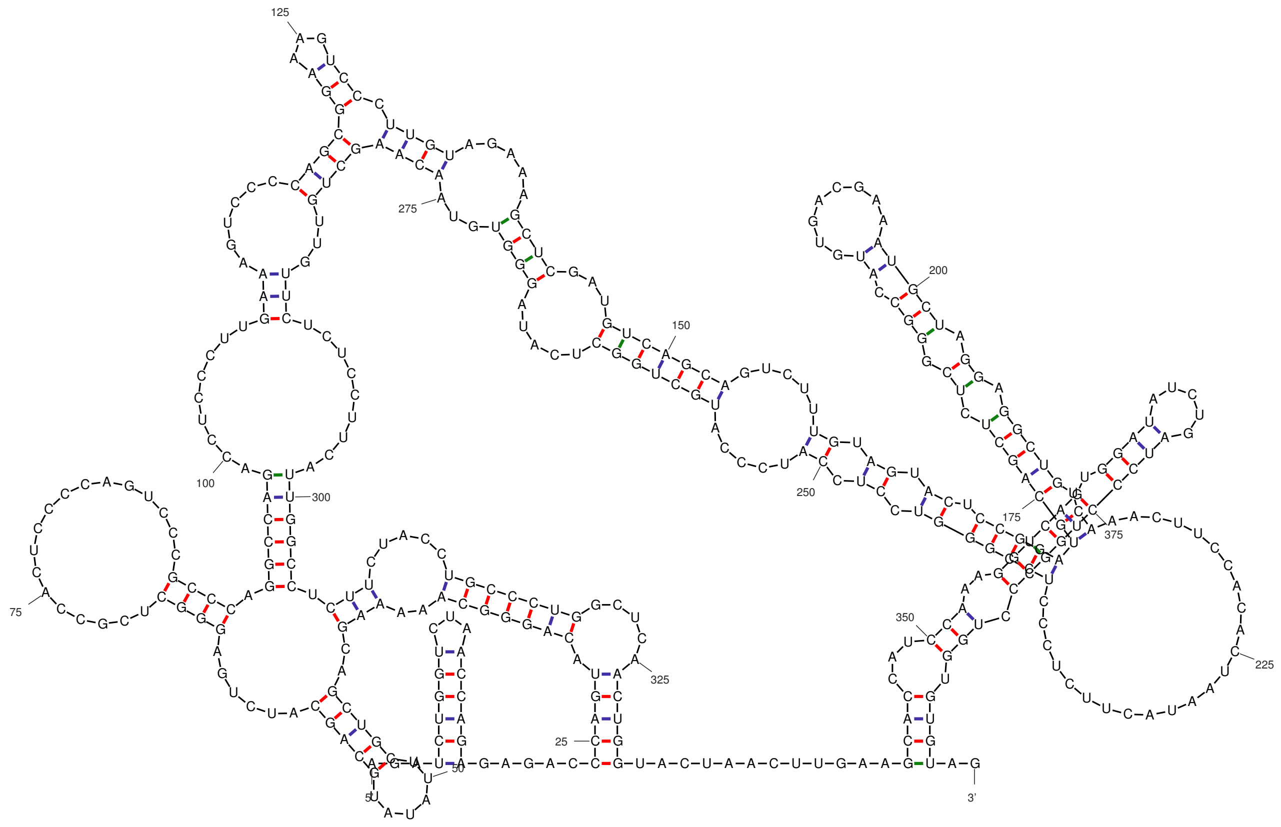

dG = -99.2   HAAmiRNA

Output of sir_graph
by D. Stewart and M. Zuker

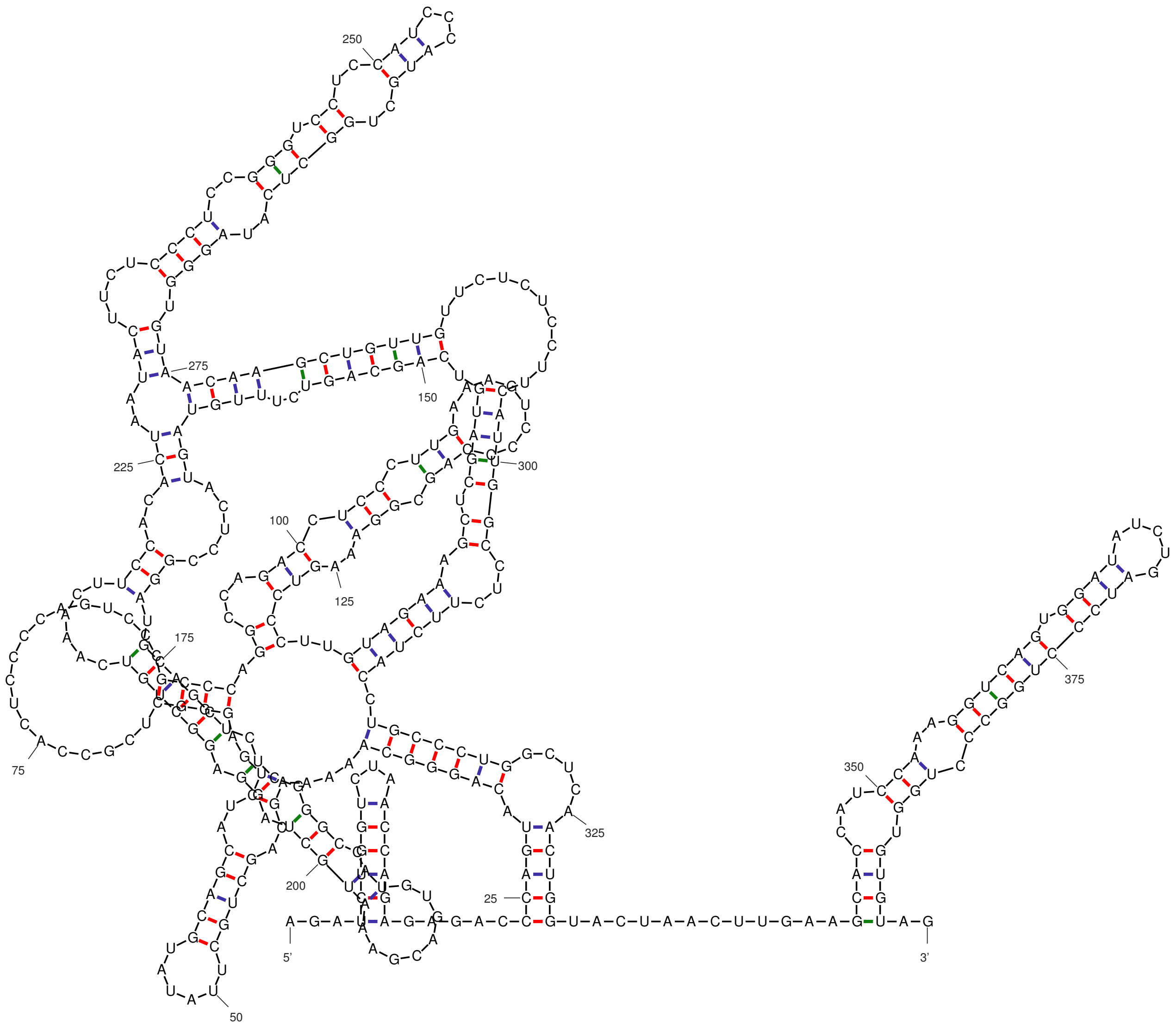

dG = -98.5   HAAmiRNA

Output of sir_graph
by D. Stewart and M. Zuker

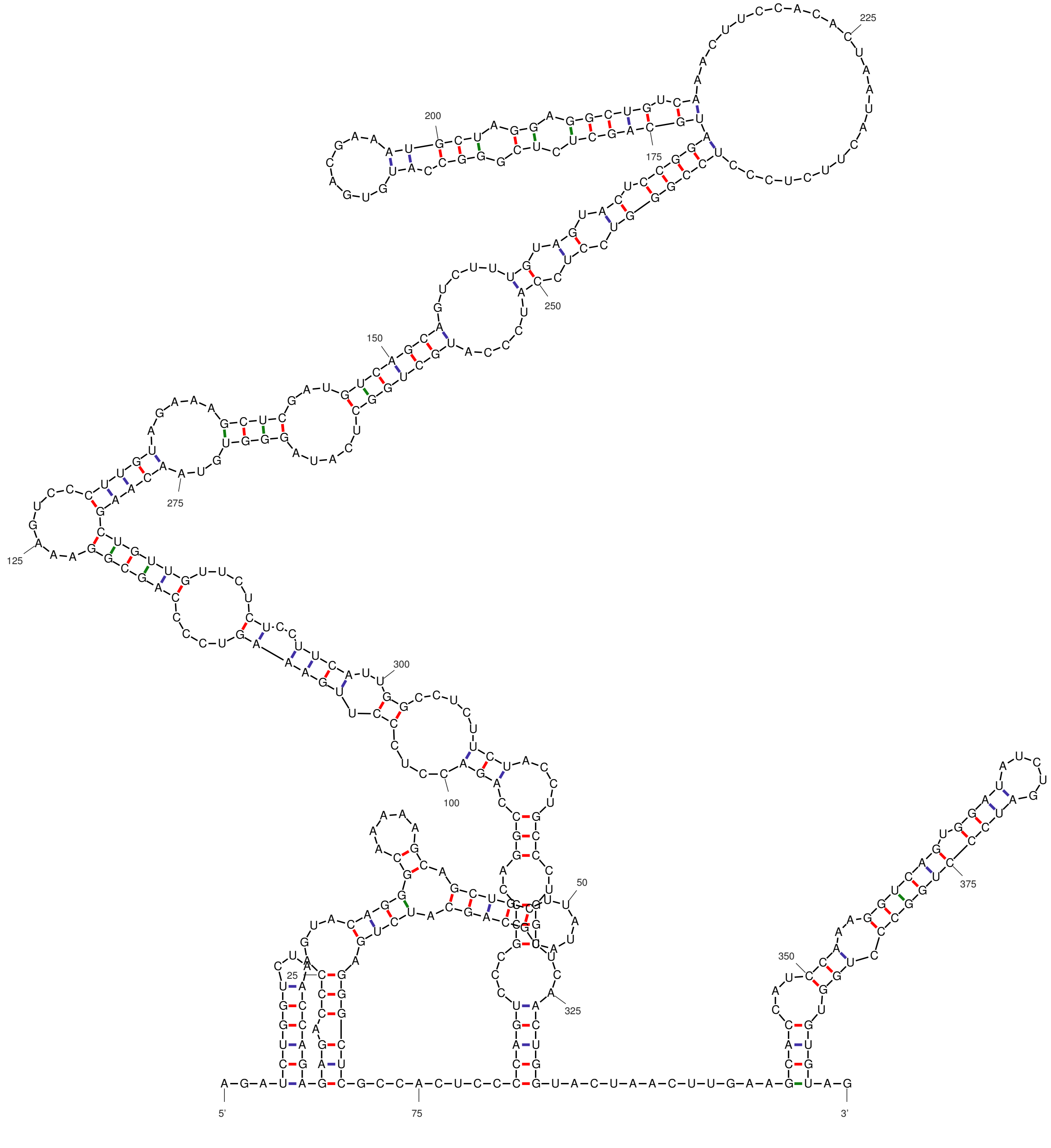

dG = -98.4   HAAmiRNA

Output of sir_graph
by D. Stewart and M. Zuker

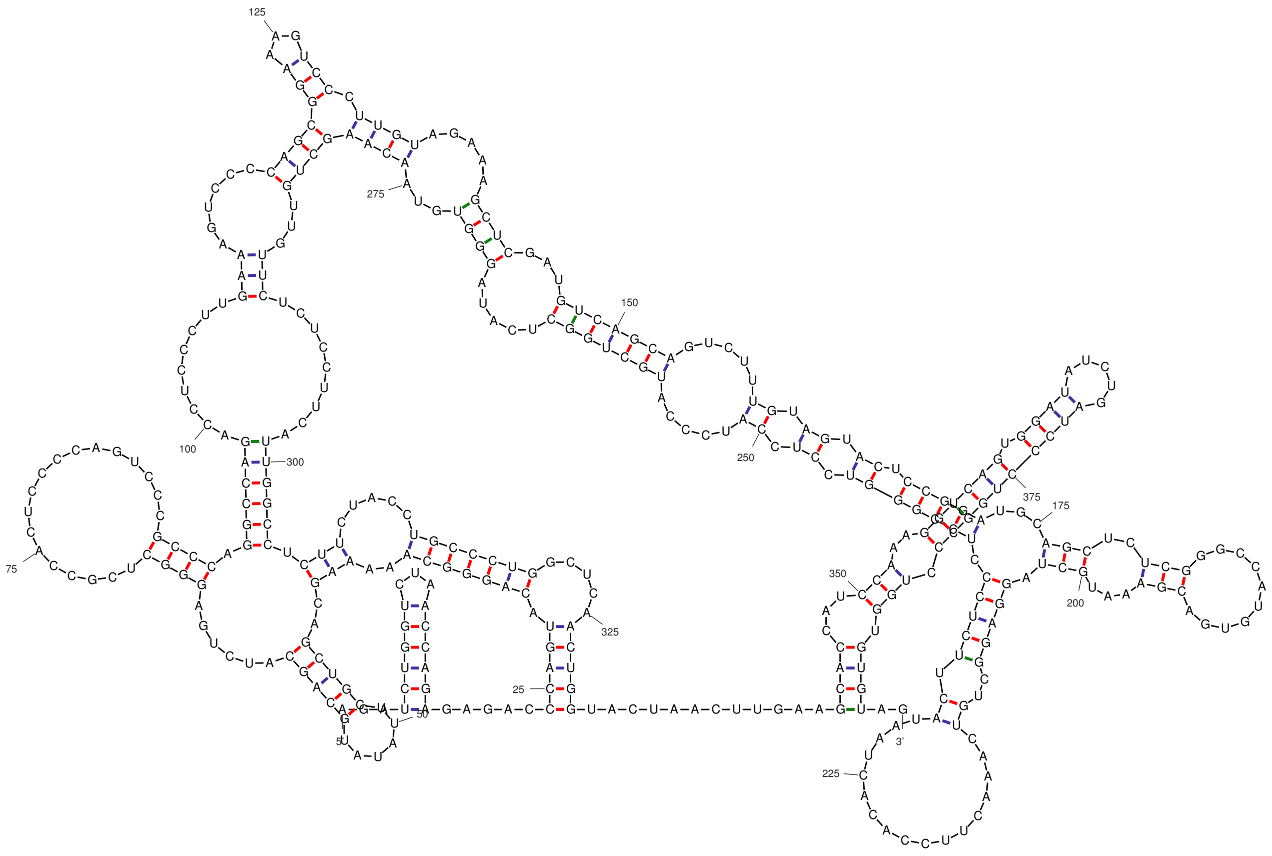

dG = -98.4   HAAmiRNA

Output of sir_graph
by D. Stewart and M. Zuker

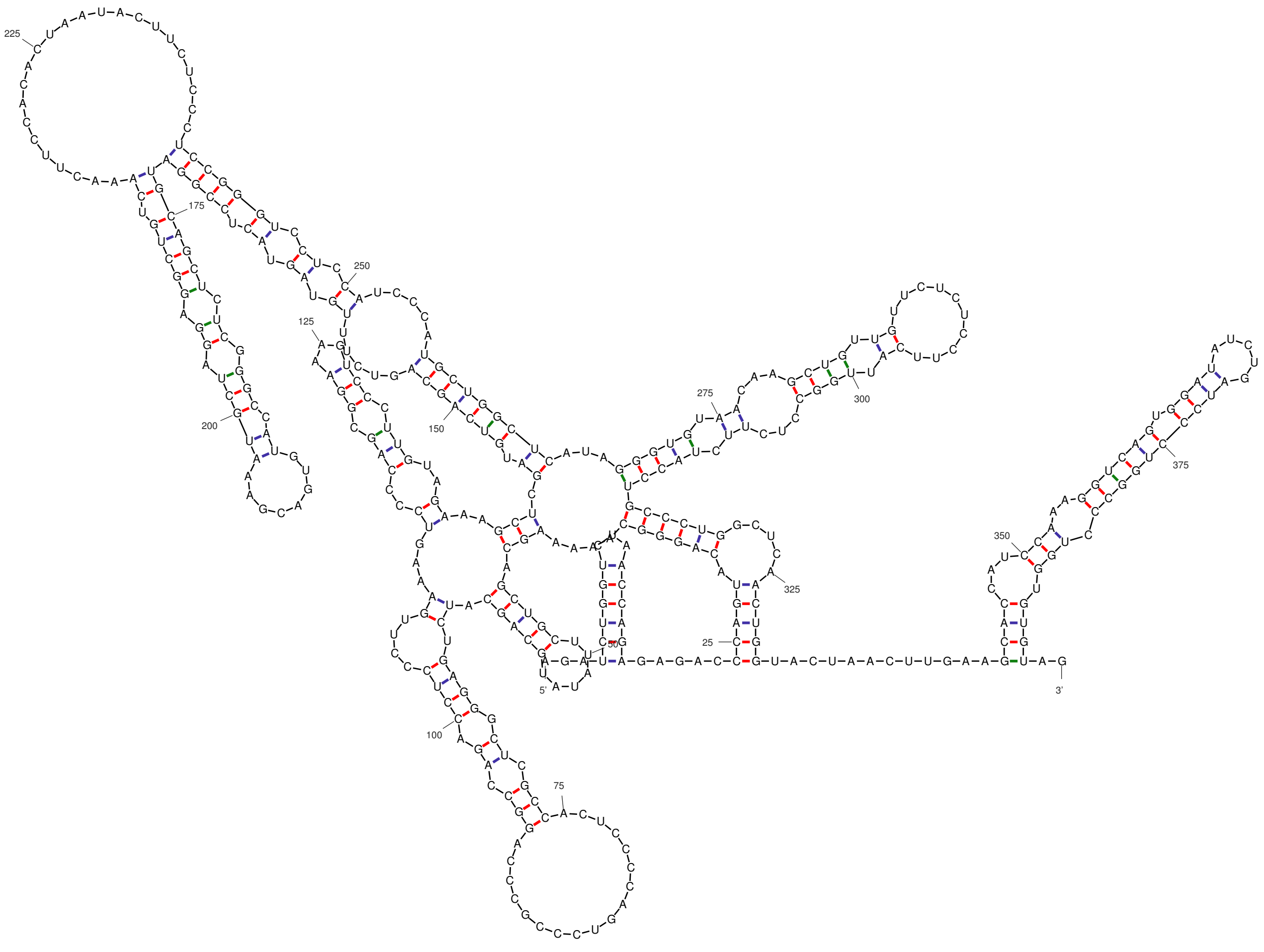

dG = -98.2 HAAmiRNA

Output of sir_graph
by D. Stewart and M. Zuker

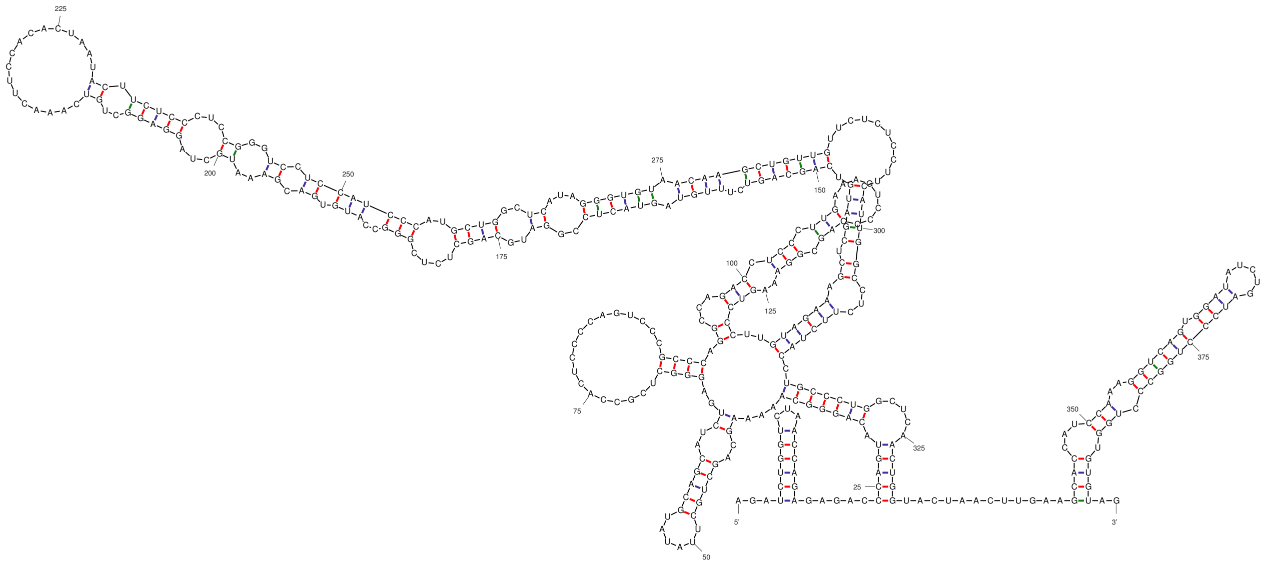

dG = -98.2 HAAmiRNA

Output of sir_graph
by D. Stewart and M. Zuker

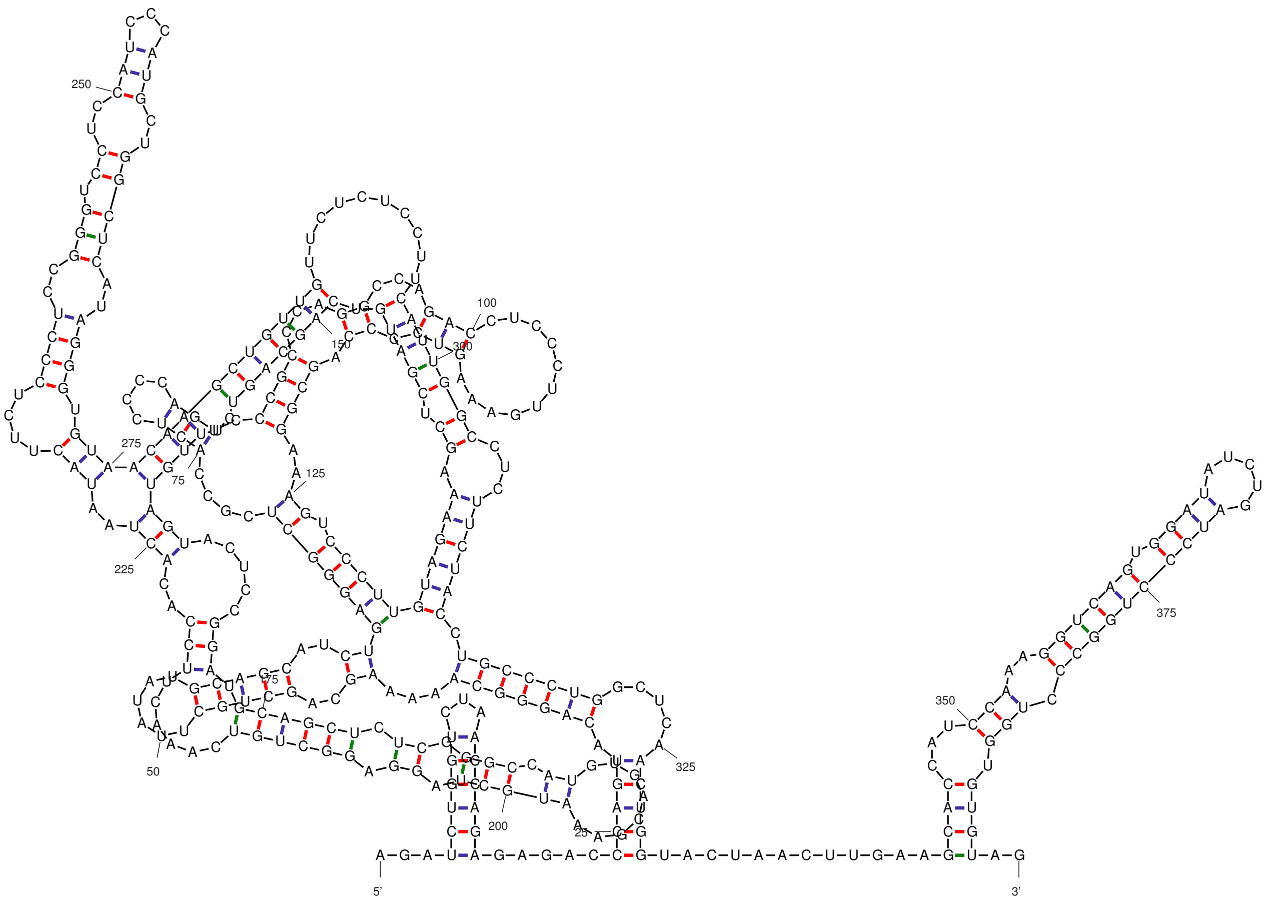

dG = -98.1 HAAmiRNA

Output of sir_graph
by D. Stewart and M. Zuker

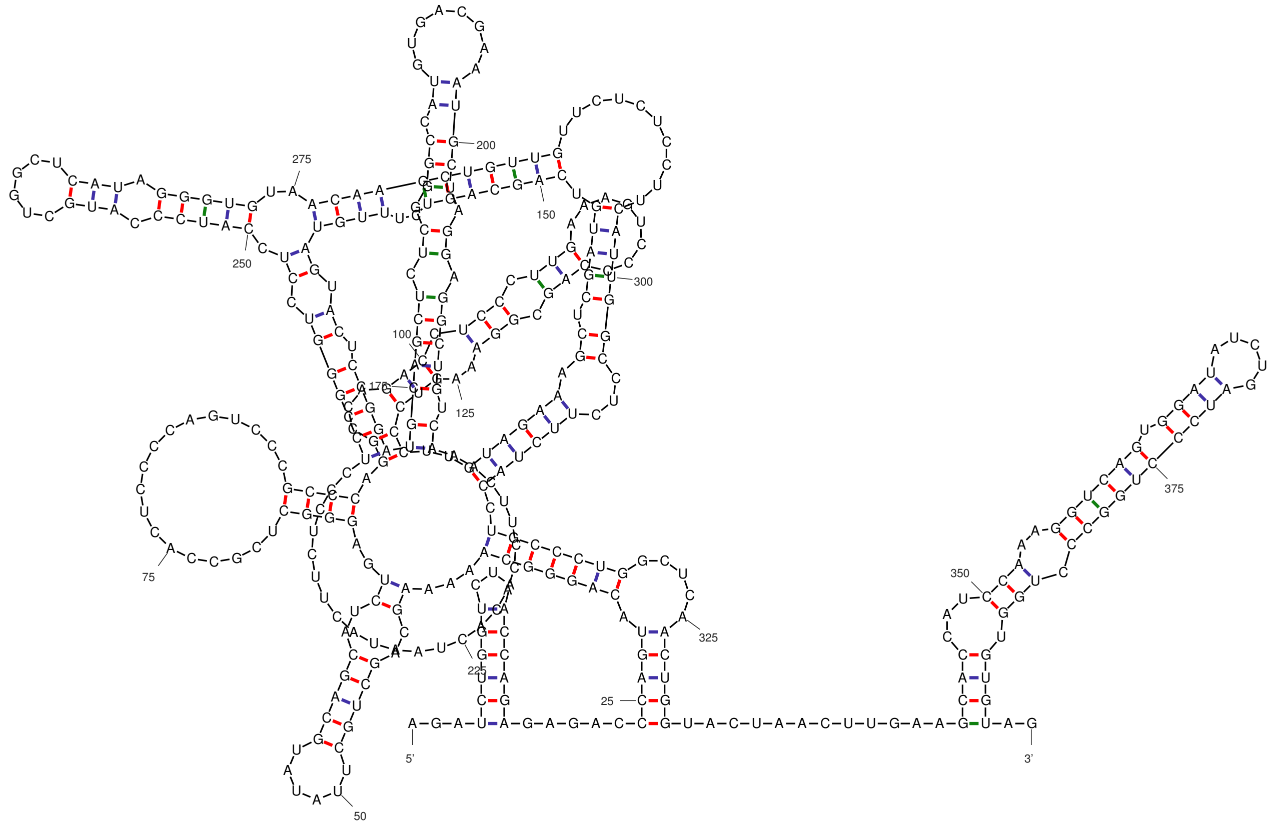

dG = -97.1   HAAmiRNA

Output of sir_graph
by D. Stewart and M. Zuker

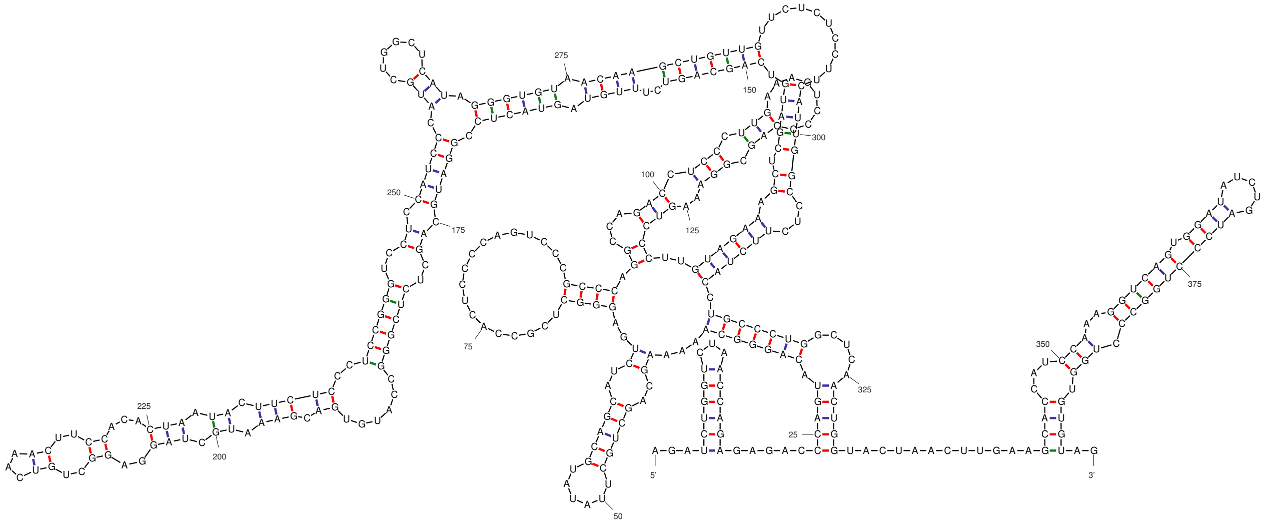

dG = -96.7   HAAmiRNA

Output of sir_graph
by D. Stewart and M. Zuker

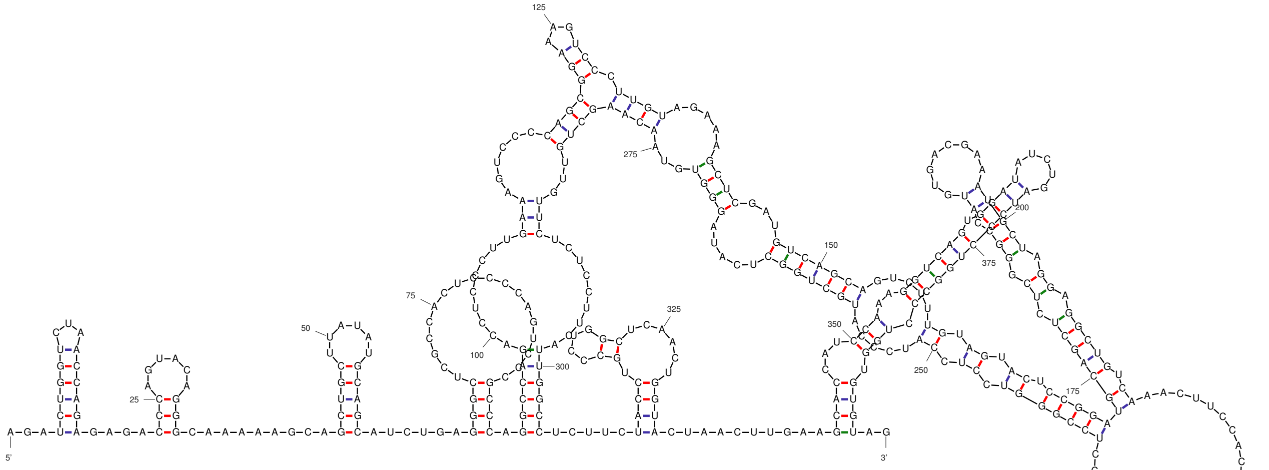

dG = -96.5   HAAmiRNA

Output of sir_graph
by D. Stewart and M. Zuker

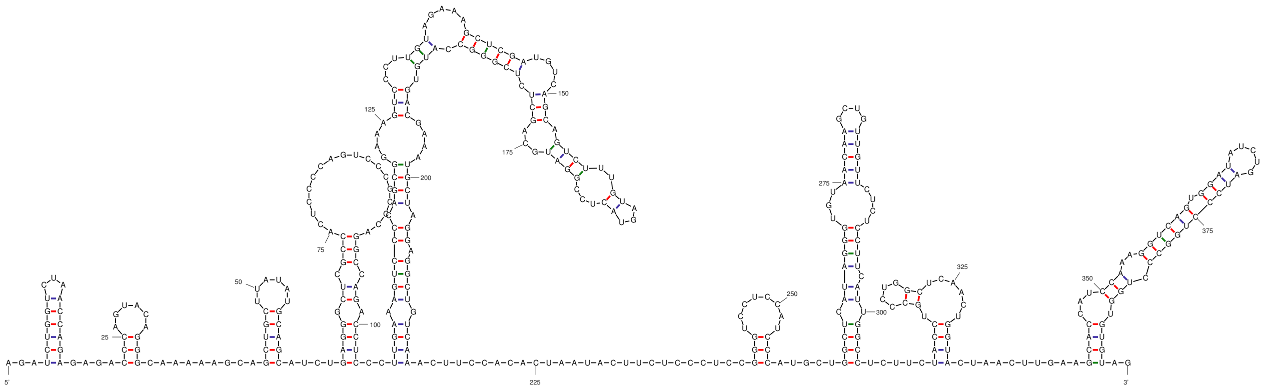

dG = -95.9   HAAmiRNA

Output of sir_graph
by D. Stewart and M. Zuker

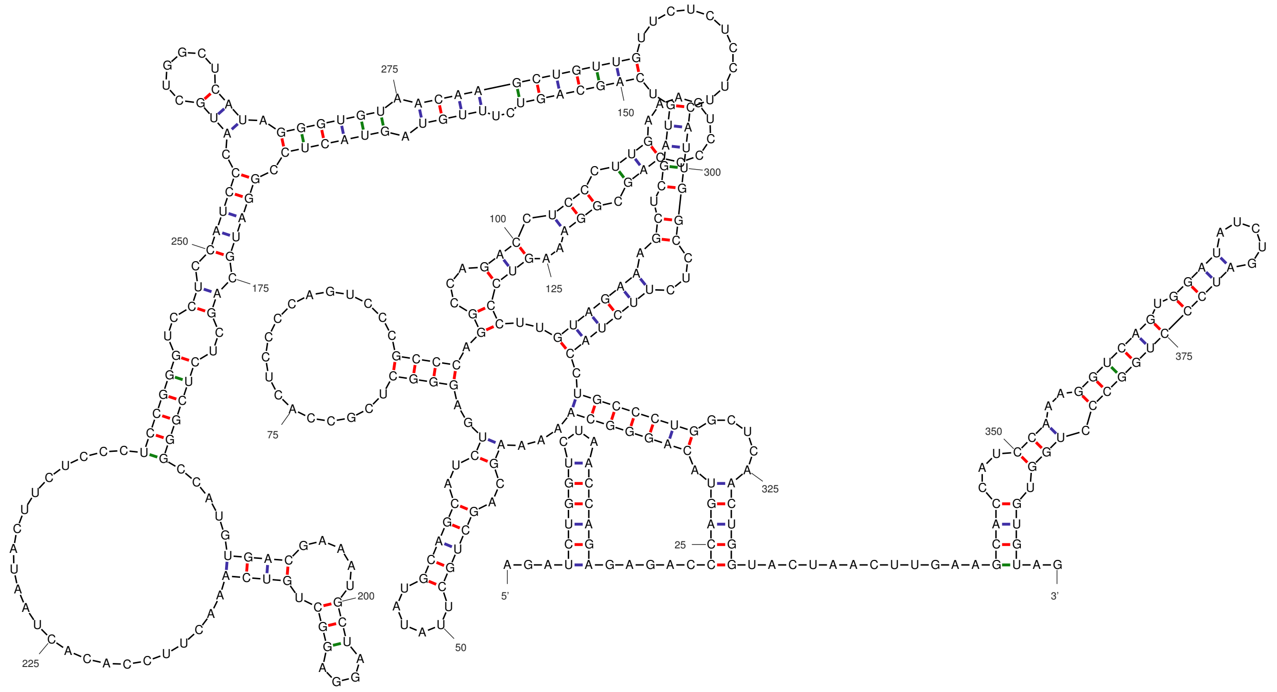

dG = -95.6   HAAmiRNA

Output of sir_graph
by D. Stewart and M. Zuker

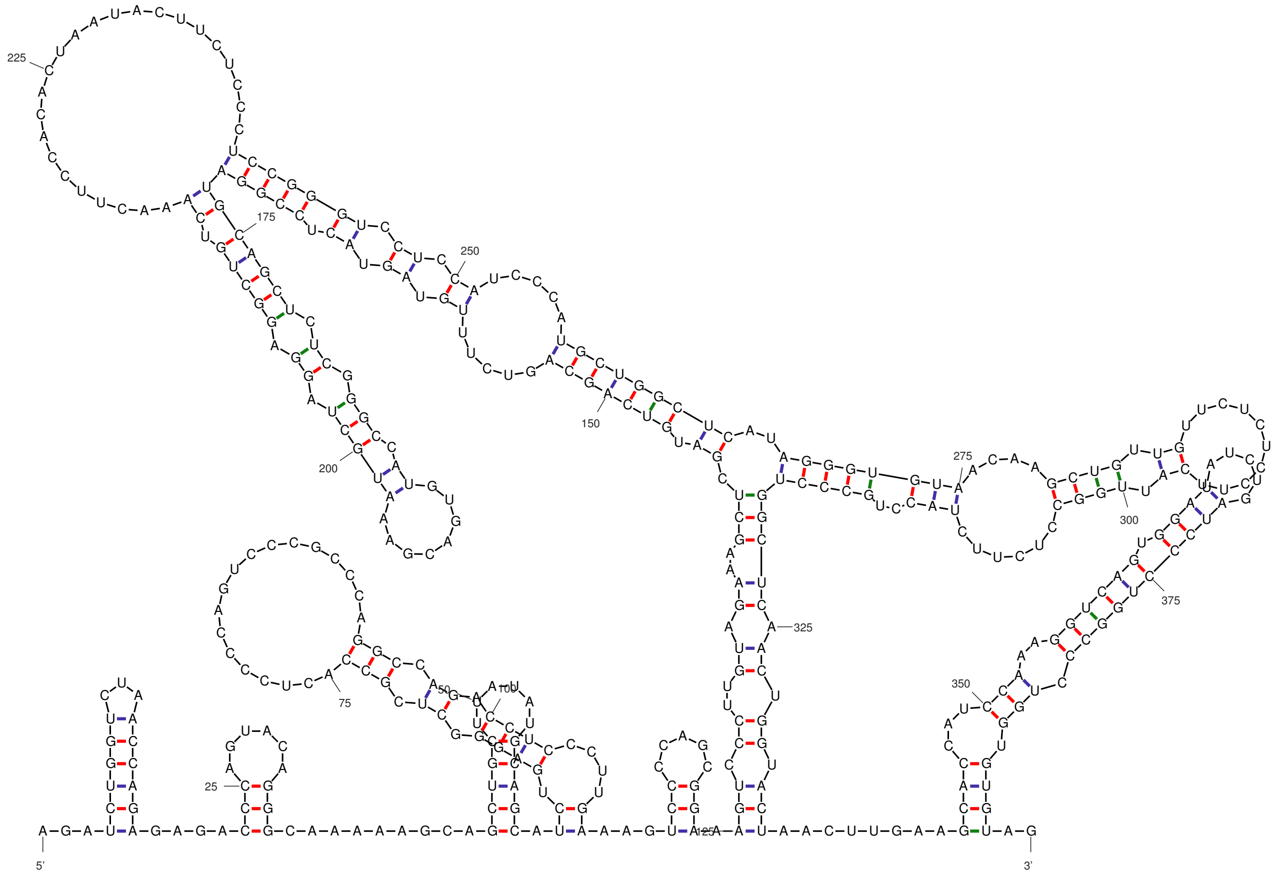

dG = -95.6 HAAmiRNA

Output of sir_graph
by D. Stewart and M. Zuker

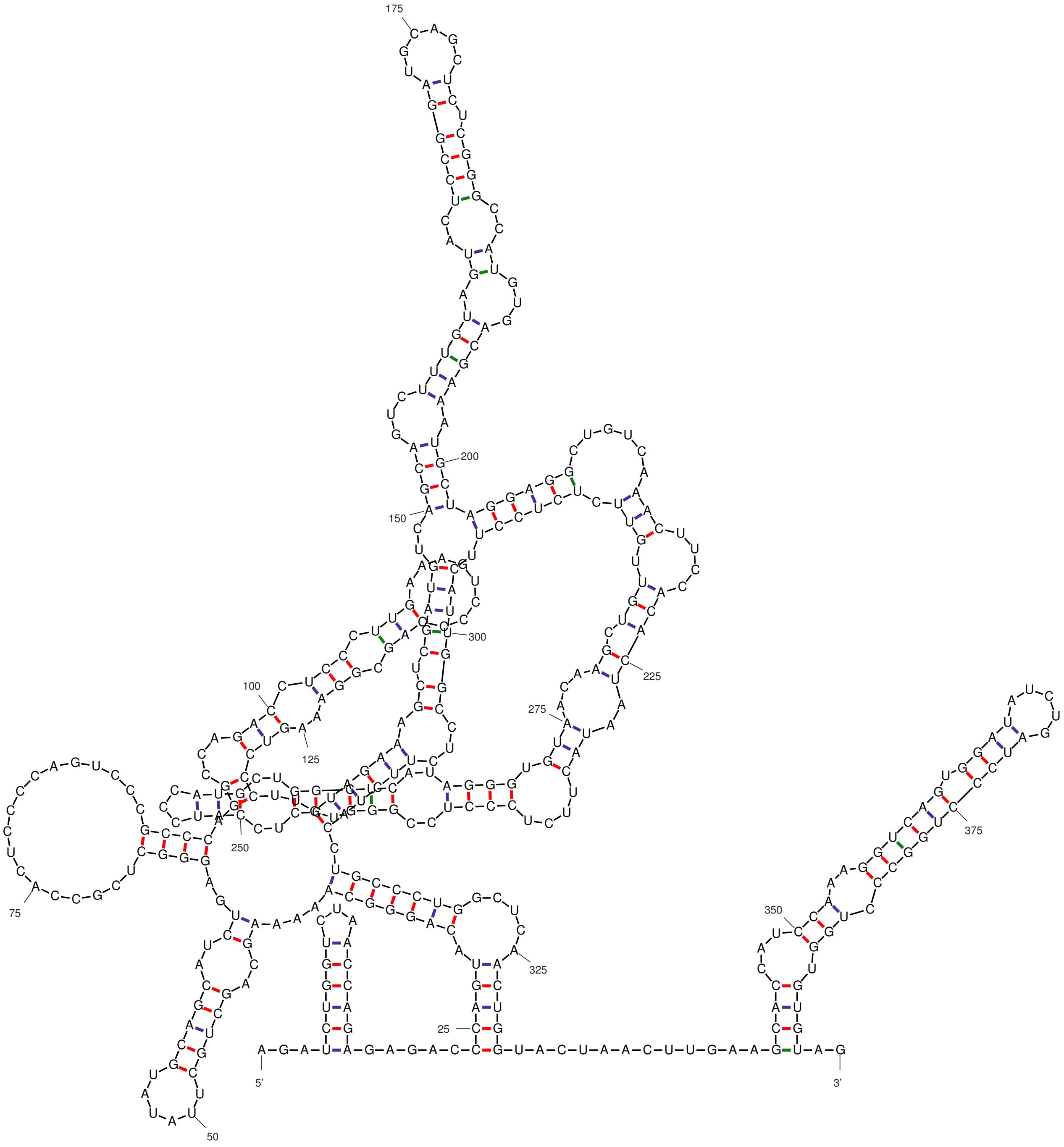

dG = -95.4 HAAmiRNA

Output of sir_graph
by D. Stewart and M. Zuker

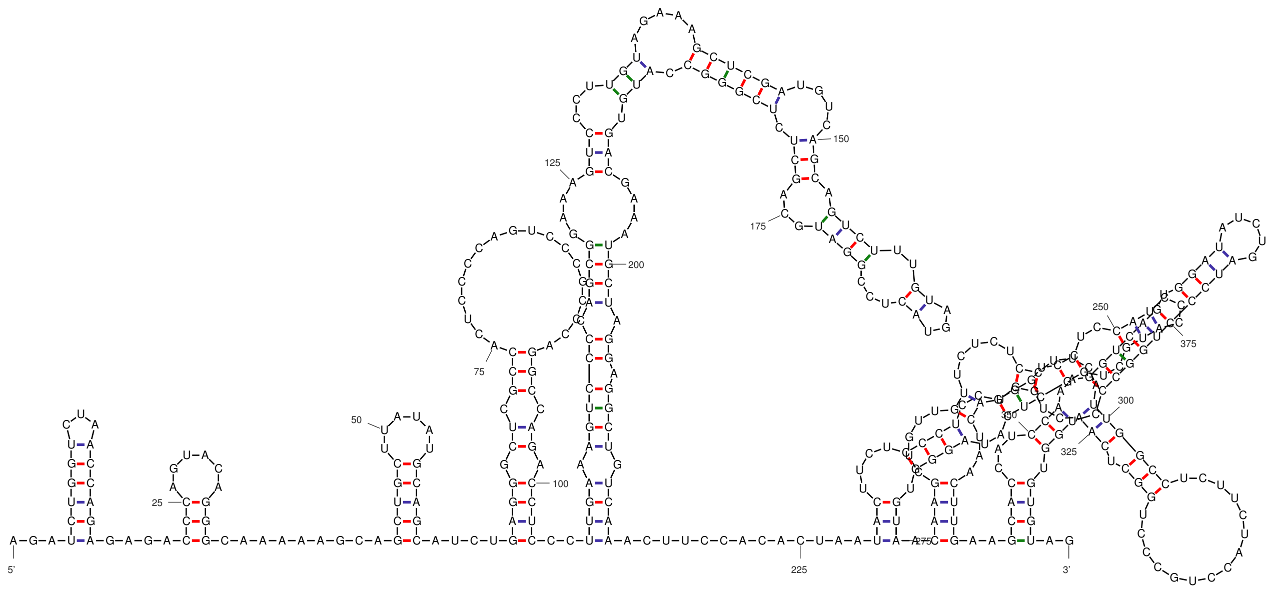

dG = -95.3   HAAmiRNA

Output of sir_graph
by D. Stewart and M. Zuker

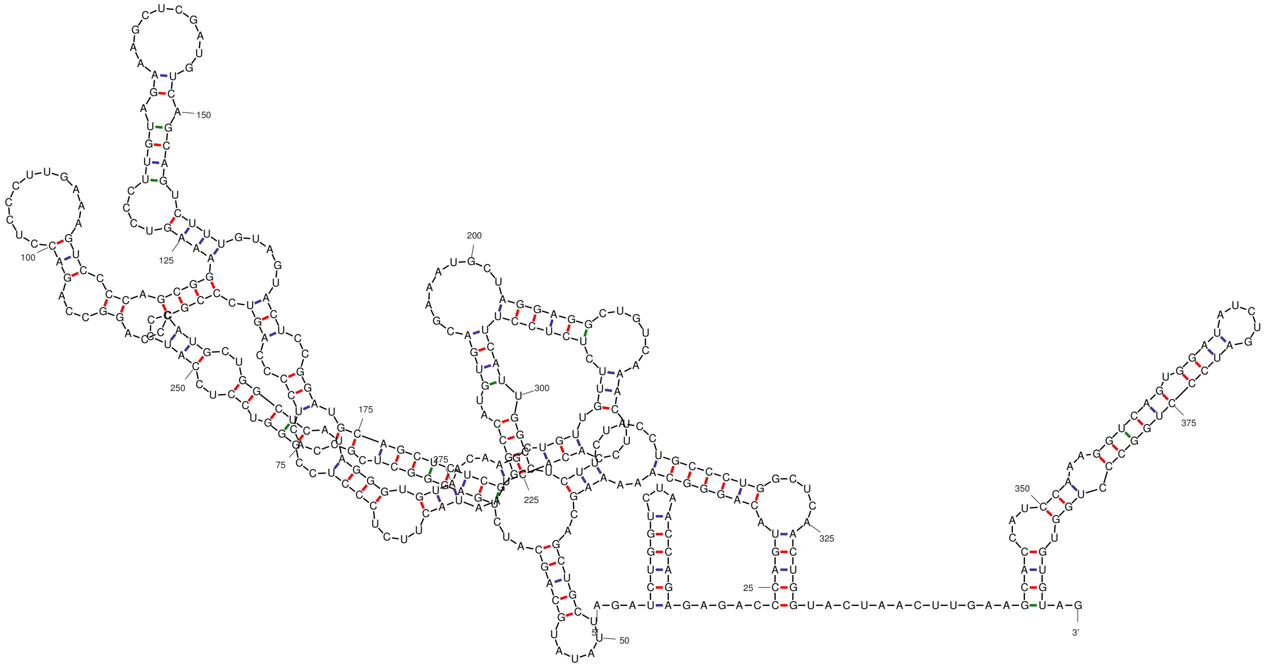

dG = -95.2 HAAmiRNA

Output of sir_graph
by D. Stewart and M. Zuker

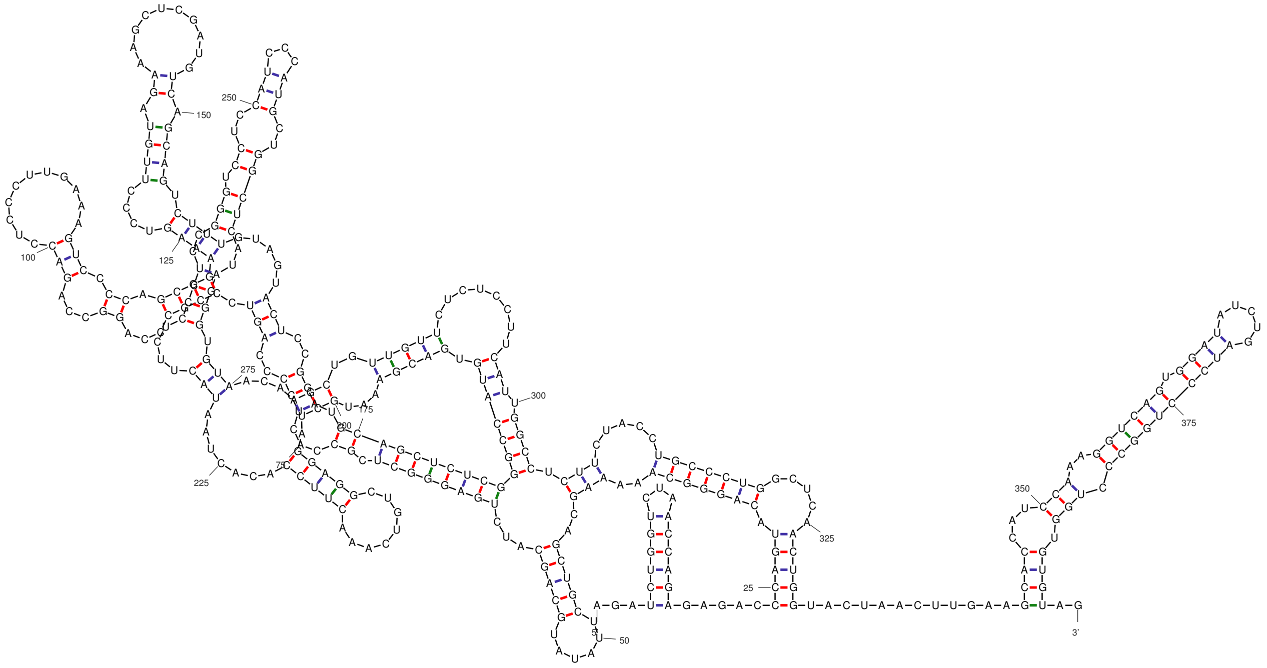

dG = -95.1 HAAmiRNA

Output of sir_graph
by D. Stewart and M. Zuker

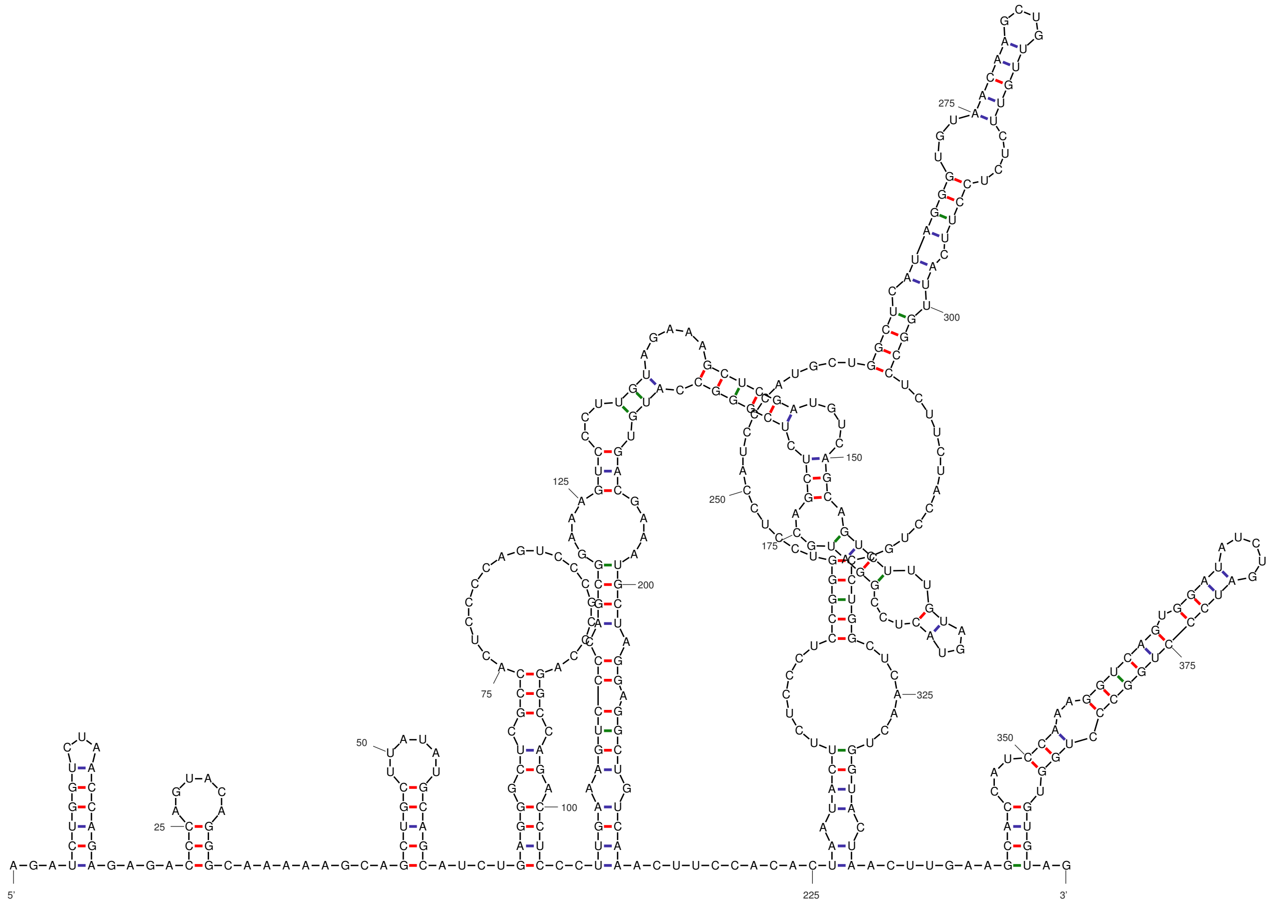

dG = -95   HAAmiRNA

Output of sir_graph
by D. Stewart and M. Zuker

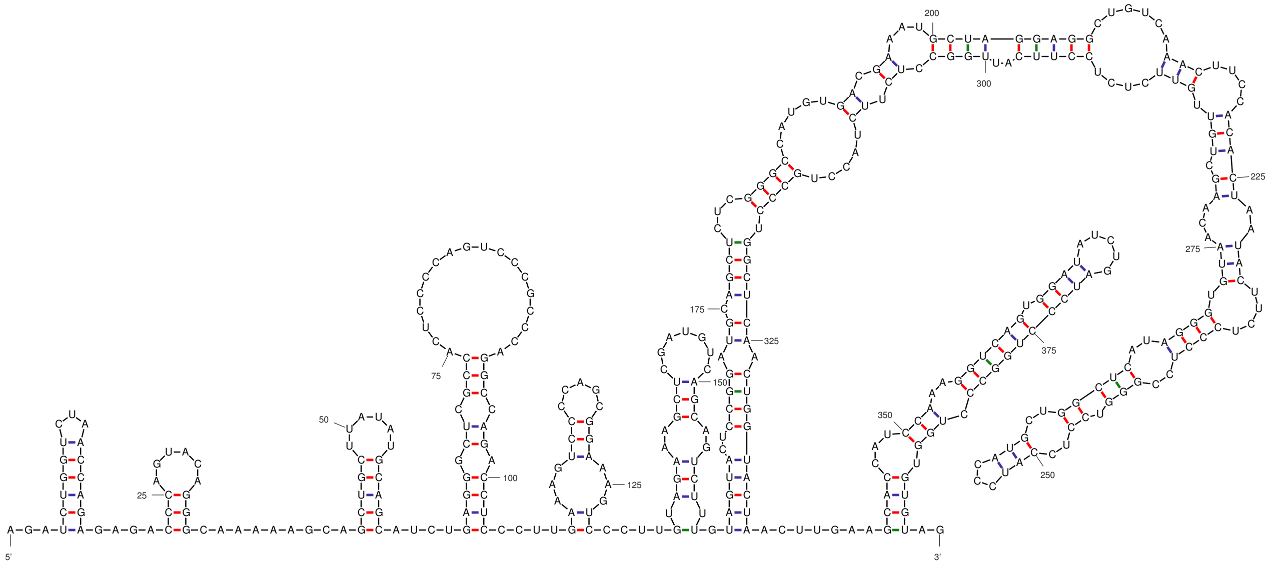

dG = -94.9   HAAmiRNA
