# Supplementary material for: RNA silencing and HIV: A hypothesis for the etiology of the severe combined immunodeficiency induced by the virus
Source: Retrovirology. 2008 Sep 11;5:79. doi: 10.1186/1742-4690-5-79 (PMC2553099; doi:10.1186/1742-4690-5-79)
Supplement: Additional file 2 — HIVaINR antisense RNA [14] and predicted HAAmiRNAs at sites 1, 2 and 3 (backward yellow arrows) are shown above the corresponding complementary sequence in the HIV-1 sequence alignments for group M (strains A, B, C, D, F1, G, H, J and K), group N, and group O, as well as chimpanzee (CPZ) strains in the long terminal repeat (LTR), as obtained from the HIV Sequence Compendium 2000[107]. HAAmiRNA 3 overlaps and is partially complementary to the nef microRNA:miR-N367 described by Omoto, S., et al., [29] and HAAmiRNA 1 and precursor (pre-HAAmiRNA 1) overlaps and is complementary sequence to the predicted #4 microRNA precursor described by Bennasser, Y. et al [30]. Predicted seed regions for the microRNAs are shaded, and sequence targeting human mRNA is underlined. The B clade HXB2 is the reference sequence, with identical sequences in the strains below, unless otherwise indicated [107]. [file 1742-4690-5-79-S2.pdf]

PREDICTED HAA miRNA 1

LBLudwig

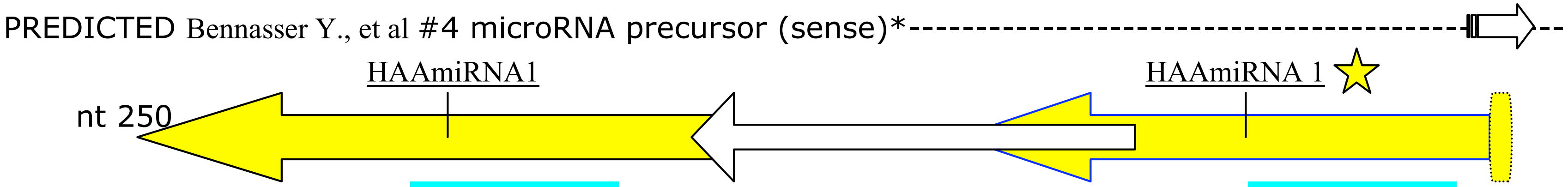


```
3' ACCUCCUGGGCCUCCCUCUUCAUAAUC 5'.......................3'CGGUGGAUCGUAAAGCAGUGUACC5'
B.TGGGATGGATGACCCGGAGAGAGAAGTGTTAGAGTGGAGGTTTGACAGCCGCCTAGCATTTCATCACGTGGCC9381
AU455  AG  A        TGAA     A            AT          A           TAC     G       AA  A     AGA    T
C2220   A        G    TGAA    C        A     A        A           T   A          CGCAGA     A
D84ZRO85         G     GA        AG          T          A     A       A   A          G  G     AA
F1VI850          G    TGAA    C               GAG       A              A       G     C  GAGA     A   A
GSE6165          G     GAA    C               G   T       A           TA             CGGAGA     A   A
H90CF056         G    TGAC  G                 GAT       AA          T    A            GACA      T
JSE7887          G    TGAA    A                  C        A             TC            CGGAGA     A   A
KMP535           G    TGAAC  C             A     AT       A           TTC             CGAAGA        A
01        A   A  G     GAA    A            C  GAT       A           TGC             CGAA   A       A
02        A      G     GAC    T            C   GATC       A          TA   A    G       CAGA      ACA    T
03        A            TGA     A   A            AT        A           T          G    TC  AACA     AGA
04        A      G     TGAA                     A         A           T          G      ACA   G      A
N.YBF30AGCA            TGATC  T   A             G   T     C  A        TC              AGAAGA   T    A    A
O.MVP5180GCT  G        TG   AC  CG G              A   A    CA       T  ATCAT      GC    AACA    T A A
O.ANT70 AT  T    A     TA   AC  T   A        A   AC   GAT          A     T   ATCT      GCAACACC   T    T    T
CPZ.CPZUS        A     TGAAC  TG  C            C       ATC      CA        CTGAA     G   TCGGAGA     AGA    T
CPZ.CPZGAB       A     TGA      C   A      G     C    G   TC      C   C       A  G    G    C    AAGA   TA  T
CPZ.CAM3         A     TG  AC  TG           C     G   TC          A        A  G    G    C    AAGA    A   A
CPZ.CAM5         G     TGAAC  TG            C          TC          A        A  G    G   TC   GA   A    C   A
CPZ.CPZANTT  GA           AC  T   A      GA    C       AGA      GAA      TGCAA       GATGAGAAGA    A   T     A
```

PREDICTED HAAmiRNA2 lbludwig

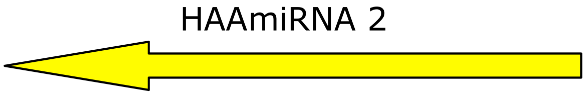


```
        3'GGUUACUUCCUCUCUUGUUGUCG  AACAAUGU5'271 HIVaINR antisense RNA
B.HXB2   CAATAAAGGAGAGAACACCAGC…TTGTTACACCCTGTGAGCCTGCAT9310
AU455     T  C  GG                     A      T    C AC             A  AT       AA(224 5'LTR)
C2220            G                     A  T            C  G              C
D84ZRO85      C G            AG        A  T            C                A  CT       A
F1VI850          G                     A  T                            CA            AA
GSE6165                                A      T    C   A               CA  CT        A
H90CF056         G                     A               C               CA
JSE7887     T    G            A        A  T        C                   CA  AT        A
KMP535       CAG               G       A  T        C                   CA  A  A      A
01              C             A        A  T        C                   CA            A
02               G                     A      T       A                CA  CT  T   AA
03               CAG  G                A           C   A                A  AT       AA
04               C   G                    T  T          C  G            A   A        A
N.YBF30  T       G            C       ATGC       C   C                 CA   AT  T   AA
O.MVP    GGG      TAC  A   TG   AGATGCTAGTC TC            T     A   CTT   TAAT
O.ANT70 AGGA   TAC    TGTG   G  GGGCTAATC  CC G           T     A   CAT  TGCC
CPZ.CPZUS                     C    A     TA     C    C G           CA     T      A
CPZ.CPZGAB  G               T        A  T          C       G      T    CA   AT  T  A
CPZ.CAM3     G              T     T  A     T       C   AC G           CA   CT       A
CPZ.CAM5    CG             C   C     AT   TA      C     C  T           CA   AT       AA
CPZ.CPZANTXXXX  AT            GG   A     TA     C   CC  C                CTT   TAC
```

PREDICTED HAAmiRNAs IN HIV LTR

lbludwig

--S.OMOTO, et.al.*---(sense) nef micro RNA: miR-N367--

HAAmiRNA 3

```
5'LTR  81
3'LTR  9165                                          3'CUAUAGGUGACUGGAAACCUACCACG 5'341 HIVaINR asRNA
B.HXB2TAGCAGAACTACACACCAGGGCCAGGGGTCAGATATCCACTGACCTTTGGATGGTGCTACAAGCTAGTAC
AU455    G         T                      AA          C       A    A                           9236 3'
C2220    G     A                    A                                          T
D84ZRO85       A                           A               T  A          G     T  G
F1VI850 G                                  A                           G       T
GSE6165G                 G                 ACT        TC    C                  T     A
H90CF056                                    AG        T     C            G     T
JSE7887 G      A                           A           C     T      A
KMP535G       A                            A           C                 G
01       G    A                            A          TC         TGT           T
02       G                                 ACT        TC                 G     T     A
03       G      XXXXXXXXXXXXXXXX           A  T       TC        A    A   G
04       G G  T                     A       AG        TC         TG            T
N.YBF30G      T                           AA T         C    CG A               T     A
O.MVP5180      TGT               G  A     ACCT        TC          A           TG TT    A    G
O.ANT70G                            A     AAC       G TC          A           TG TT    A
CPZ.CPZUS         T       C       T  A   A                C  A   A A   G         TT     T
CPZ.CPZGAB                 A     A       AACA         TC       CTGT              T     A
CPZ.CAM3                                  A            C       A   A  A  GG      TT    A    G
CPZ.CAM5          T                       A             C      A   A             T          G
CPZ.CPZANT        T         GA   A        A  T                 CTGTAGA        T  TT     T
```
